# Using long-read CAGE sequencing to profile cryptic-promoter-derived transcripts and their contribution to the immunopeptidome

**DOI:** 10.1101/gr.277061.122

**Published:** 2023-12

**Authors:** Ju Heon Maeng, H. Josh Jang, Alan Y. Du, Shin-Cheng Tzeng, Ting Wang

**Affiliations:** 1 Department of Genetics, Washington University School of Medicine, St. Louis, Missouri 63110, USA;; 2 Edison Family Center for Genome Sciences and Systems Biology, Washington University School of Medicine, St. Louis, Missouri 63110, USA;; 3 Donald Danforth Plant Science Center, St. Louis, Missouri 63132, USA;; 4 McDonnell Genome Institute, Washington University School of Medicine, St. Louis, Missouri 63108, USA

## Abstract

Recent studies have shown that the noncoding genome can produce unannotated proteins as antigens that induce immune response. One major source of this activity is the aberrant epigenetic reactivation of transposable elements (TEs). In tumors, TEs often provide cryptic or alternate promoters, which can generate transcripts that encode tumor-specific unannotated proteins. Thus, TE-derived transcripts (TE transcripts) have the potential to produce tumor-specific, but recurrent, antigens shared among many tumors. Identification of TE-derived tumor antigens holds the promise to improve cancer immunotherapy approaches; however, current genomics and computational tools are not optimized for their detection. Here we combined CAGE technology with full-length long-read transcriptome sequencing (long-read CAGE, or LRCAGE) and developed a suite of computational tools to significantly improve immunopeptidome detection by incorporating TE and other tumor transcripts into the proteome database. By applying our methods to human lung cancer cell line H1299 data, we show that long-read technology significantly improves mapping of promoters with low mappability scores and that LRCAGE guarantees accurate construction of uncharacterized 5′ transcript structure. Augmenting a reference proteome database with newly characterized transcripts enabled us to detect noncanonical antigens from HLA-pulldown LC-MS/MS data. Lastly, we show that epigenetic treatment increased the number of noncanonical antigens, particularly those encoded by TE transcripts, which might expand the pool of targetable antigens for cancers with low mutational burden.

Immunopeptidome profiling using liquid chromatography with tandem mass spectrometry (LC-MS/MS) is widely used to identify cancer-specific antigens for targeted immunotherapy (Chong et al. 2020; Arnaud et al. 2022). The current proteomics workflow detects peptides by comparing spectra from LC-MS/MS to predicted spectra of in silico digested peptides from the reference proteome database. As a result, identified peptides are currently limited to the composition of the reference proteome database (Purcell et al. 2019; Chong et al. 2020). Recent research expanded the proteome database to include unannotated proteins by mining genomics data, primarily RNA-seq, which revealed peptides that went undetected owing to the limitations of conventional reference proteome approaches (Laumont et al. 2016; Attig et al. 2019; Chong et al. 2020; Cuevas et al. 2021). Thus, accurate and comprehensive transcriptome profiling can be instrumental to the increase of power of LC-MS/MS analysis.

Activation of transposable elements as alternate promoters to protein coding genes can modify downstream exons and produce unannotated amino acids (Wiesner et al. 2015; Scarfò et al. 2016; Brocks et al. 2017; Jang et al. 2019; Shah et al. 2023). Although most TEs are epigenetically repressed in somatic tissues, some TEs are exapted to function as promoters in a tissue-specific or developmental stage–specific manner (Bourque et al. 2018; Modzelewski et al. 2021), upon extrinsic cues (Brocks et al. 2017; McDonald et al. 2021), or in diseases, such as cancer and autoimmunity, as a result of aberrant epigenetic regulation (Babaian and Mager 2016; Chuong et al. 2017; Bourque et al. 2018). For instance, Jang et al. (2019) discovered that an *Alu*Jb alternate promoter drives expression of a chimeric LIN28B protein prepended with unannotated 22 amino acids, leading to an up-regulated *LIN28B* oncogene in many cancers. Several recent studies included endogenous TEs and TE transcripts as an additional source for discovering antigens (Attig et al. 2019; Kong et al. 2019; Chong et al. 2020; Bonté et al. 2022; Shah et al. 2023). Because the transcriptional reactivation of TEs is widespread in cancer (Jang et al. 2019; Shah et al. 2023) and further stimulated by epigenetic therapy (Brocks et al. 2017; Jones et al. 2019; Kong et al. 2019), investigating TE transcripts as an underappreciated source of antigens holds a strong promise to enhance cancer immunotherapy, especially for cancers with a relatively low mutation load.

To investigate TE transcripts as a source of cancer antigens, it is important to accurately define the structure and sequence of the transcripts, especially their 5′ end, where noncanonical ATGs may generate novel peptides. Cap Analysis of Gene Expression (CAGE) and its variations, including nanoCAGE, have been widely used to detect transcription start sites (TSSs) by the FANTOM Consortium (Plessy et al. 2010; The FANTOM Consortium and the RIKEN PMI and CLST (DGT) 2014; Ramilowski et al. 2020). However, these libraries sequenced using short-read technologies are not optimized for detecting TE transcripts. First, sequencing reads from short-read technologies can align to multiple genomic loci equally well (Lee and Schatz 2012; Sexton and Han 2019), a phenomenon known as multimapping reads (Conesa et al. 2016). The mapping ambiguity often results in discarding such reads, leading to reduced recall in identifying promoters and TSSs from regions with low mappability scores whose sequences are repetitive in nature and often derived from TEs (Conesa et al. 2016). Second, the ability to precisely construct previously uncharacterized transcript structure is highly dependent on read length and read coverage of the transcript (Steijger et al. 2013; Pertea et al. 2015). Therefore, we need both CAGE-seq and RNA-seq and additional complex algorithms to assemble previously uncharacterized transcripts for the purpose of identifying open reading frames and predicting unannotated peptide sequences (Boley et al. 2014). These methods are often ad hoc, with highly variable performance, and are difficult to benchmark (Engström et al. 2013; Steijger et al. 2013; Conesa et al. 2016).

Long-read technology of Pacific Biosciences (PacBio) SMRT sequencing or Oxford Nanopore sequencing can be coupled with CAGE to tackle these challenges. Moore et al. (2022) showed that TSS peaks by PacBio Iso-Seq are largely concordant with TSS peaks called by RAMPAGE, CAGE-seq, and GRO-cap signals using human cancer cell lines. In addition, robust long-read full-length RNA-seq has the advantage of trivially identifying alternative transcripts. Previous studies have also shown that long-read sequencing can identify thousands of uncharacterized transcript isoforms (Au et al. 2013; Tilgner et al. 2015; Glinos et al. 2022), which may encode unannotated proteins (Tardaguila et al. 2018; Glinos et al. 2022) and antigens (Oka et al. 2021). One caveat of long-read RNA-seq is the lower throughput relative to short-read RNA-seq. However, the typical PacBio sequel II platform can now generate 3 million to 4 million reads per SMRT cell, which is close to the median CAGE-seq (4 million reads) that FANTOM5 used for profiling promoters (The FANTOM Consortium and the RIKEN PMI and CLST (DGT) 2014). Wyman et al. (2020) showed that 86%–88% of genes with five or more TPM in GM12878 cells are reproducibly detected using PacBio Iso-Seq data of about 3 million reads. Also, Berrens et al. (2022) showed that long-read technology aligned reads unambiguously to L1 elements in the mouse genome that were not uniquely mappable using short-read technology. Therefore, combining CAGE technology with long-read sequencing will likely provide an optimal solution to the discovery of previously uncharacterized TE transcripts for immunopeptidome analysis.

In this study, we presented computational toolkits to use long-read CAGE data for studying transcription from highly repetitive regions and its impact on immunopeptidome repertoires. We compared two long-read CAGE library construction strategies using either poly(A) priming (LRCAGE) or random hexamer priming (LRhex) with the original nanoCAGE results. Using the cancer cell line H1299 as an example, we benchmarked the sensitivity of long-read CAGE for TSSs with low mappability scores, including TEs, explored uncharacterized transcripts, and unannotated proteins. Furthermore, we investigated TE transcripts, including endogenous retroviruses (ERVs), as a source of antigens upon epigenetic treatment.

## Results

### Using long-read CAGE to detect TSSs

**Figure 1.**
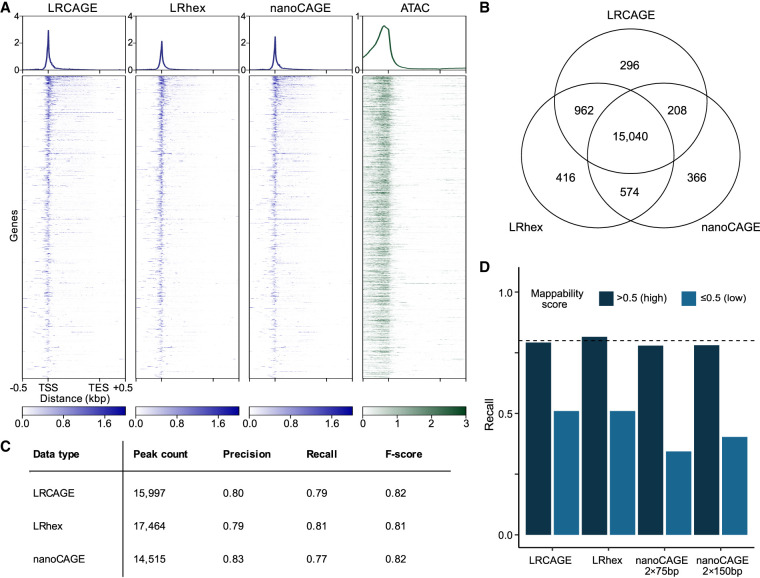
Benchmark of long-read CAGE data for promoter identification. (*A*) Heatmap of coverage across gene bodies. For coverage, 5′ ends of read were used for LRCAGE, LRhex, and nanoCAGE, and Tn5 insertion sites were used for ATAC-seq data. (TES) Transcription end site. (*B*) Venn diagram showing intersections of active GTSSs detected by LRCAGE, LRhex, and nanoCAGE (2 × 75-bp) peaks. (*C*) Peak count, precision, recall, and *F*-score by LRCAGE, LRhex, and nanoCAGE (2 × 75 bp) based on active GTSSs. (*D*) Recall as a function of mappability scores using 20,649 and 300 active GTSSs with high and low mappability scores, respectively. LRCAGE: 79% (16,353, high), 51% (153, low); LRhex: 82% (16,839, high), 51% (153, low); nanoCAGE 2 × 75 bp: 78% (16,085, high), 34% (103, low); and nanoCAGE 2 × 150 bp: 78% (16,122, high), 40% (121, low).

To examine the potential of long-read sequencing to identify TSSs, we generated LRCAGE, LRhex, and nanoCAGE libraries using the H1299 cell line. LRCAGE and LRhex are two derivatives of the PacBio Iso-Seq protocols, profiling full-length transcripts and 5′ end of transcripts, respectively. We isolated poly(A) RNA and used poly-dT primer for LRCAGE and random hexamer for LRhex to perform reverse transcription. To capture 5′ ends, we used the same nanoCAGE template switching oligos (TSOs) for all three methods (Supplemental Fig. S1A). LRCAGE sequences full-length transcripts, whereas LRhex sequences 5′ portions of transcripts (Supplemental Fig. S1B,C). One LRCAGE library and one LRhex library were sequenced using PacBio sequel II to more than 3 million reads per library. The NanoCAGE (short-read) library was made using standard protocol (Poulain et al. 2017) and was sequenced using Illumina NextSeq 500 at more than 33 million (2 × 75 bp) and more than 7 million (2 × 150 bp) reads. Reads were aligned to the hg38 reference genome using STAR (Dobin et al. 2013) for nanoCAGE and using minimap2 (Li 2018) for LRCAGE and LRhex (Methods) (Supplemental Table S1). For benchmark analysis, we used the number of deduplicated/uniquely mapped reads as the constant condition across three libraries (3 million reads), with the assumption that deduplicated/uniquely mapped reads correspond to transcript molecules. For paired-end nanoCAGE, we used read 1 of the 2 × 75-bp data unless stated otherwise. As expected, the sizes of RNA fragments mapped by long-read CAGE were longer than that of nanoCAGE (Supplemental Fig. S2A). To verify the quality of our data, we assessed the distribution of 5′ ends of reads as a function of their distance to the closest TSSs in GENCODE annotation (GTSSs). The 5′ ends of the reads were strongly enriched at the 5′ ends of annotated genes but depleted across the remaining gene body (Fig. 1A). More than 70% of the 5′ ends of the reads were located within promoters, 5′ UTRs, and first exons defined by GENCODE (Supplemental Fig. S2B). All methods showed comparable distributions, with 59%–64% of aligned reads located within a ±100-bp window of GTSSs (Supplemental Fig. S2D). Seventy-six percent of LRCAGE reads reached GENCODE-annotated TESs (GTESs) whereas only 16% of LRhex reads and 3% of nanoCAGE reads did (Supplemental Fig. S2E).

We also assessed the relationship between CAGE peaks and transcriptionally-active GTSSs (“active GTSSs”). As a control, we defined 20,949 active GTSSs using short-read RNA-seq data (Methods). Among these, 17,862 were overlapped by the union of CAGE peaks from the three methods and defined as “detected GTSSs”; 84.2% of the detected GTSSs were shared across three peak call sets (Fig. 1B). Using nanoCAGE (2 × 150 bp) of 3 million reads and nanoCAGE (2 × 75 bp) of 22 million reads gave comparable overlaps of 84.1% and 85.7%, respectively (Supplemental Fig. S2F,G). As expected, genomic annotation of CAGE peaks showed that 82%–84% of peaks were in promoter regions, and 7% were located within 5′ UTR and first exons (Supplemental Fig. S2C). The three methods only had slight differences in precision and recall for detecting active GTSSs using a distance of 200 bp as the tolerance window (Fig. 1C). LRCAGE and LRhex had slightly higher recall but slightly lower precision compared with that of nanoCAGE. In addition, peak strengths from all three data were strongly correlated with gene expression levels based on RNA-seq (Spearman's correlation: 0.69–0.71) (Supplemental Fig. S3A,B). These correlations were comparable to the previously reported Spearman's correlation between CAGE-seq and RNA-seq (0.66) (Kawaji et al. 2014). Taken together, these results support that 5′ read ends of the LRCAGE and LRhex libraries are highly enriched in GTSSs, comparable to conventional nanoCAGE.

Having established the consistency among the three CAGE data to detect active GTSSs, we set out to assess their potential differences in detecting active GTSSs as a function of three variables: (1) expression levels, (2) mappability scores at GTSSs, and (3) transcript length (Methods). As expected, the recalls of the three data increased steadily and consistently as expression levels increased, and all reached >80% recall at ≥10 TPM (Supplemental Fig. S2H; Supplemental Table S2). Also as expected, LRCAGE and LRhex had much higher recall than nanoCAGE for the 300 active GTSSs within ≤0.5 mappability scores, whereas for the 20,649 active GTSSs within >0.5 mappability scores, the recalls of the three methods were once again comparable (Fig. 1D; Supplemental Table S2). Of 49 active GTSSs with ≤0.5 mappability scores that were missed by nanoCAGE peaks, 81% were supported using multimapping reads. For LRCAGE and LRhex, the lower recall for active GTSSs with ≤0.5 mappability scores compared with GTSSs with >0.5 mappability scores was largely owing to segmental duplications (at least 5 kbp long and having ≥99% sequence identity) (Bailey et al. 2002). These duplicated genomic regions are longer than the length of long reads and, thus, are not uniquely mappable even by long reads (Wenger et al. 2019; Prodanov and Bansal 2020). For transcript length, all three data showed slightly reduced recall for long transcripts with LRCAGE recall decreasing faster as transcript length increased, possibly owing to longer RNA templates leading to incomplete reverse transcription (Supplemental Fig. S2I; Supplemental Table S2). This outcome reflected the reduced efficiency in making longer cDNAs (Prawer et al. 2023). These analyses highlight the distinct value of long-read sequencing in mapping promoters with low mappability and suggest a potential compromise between obtaining a more complete 5′ transcript and a full-length transcript.

We further examined the completeness of the two long-read methods in comparison with conventional nanoCAGE results. Using 16,188 active GTSSs detected by nanoCAGE peaks as a benchmark, we defined rediscovery rates as percentages of active GTSSs that are also detected by long-read data. Overall, the rediscovery rates of LRCAGE and LRhex were 94.2% and 96.5%, respectively, which were indistinguishable from the 95.9% of nanoCAGE pseudoreplicate (ps). The rediscovery rate was robust as a function of expression levels and mappability scores (Supplemental Fig. S4A,C). However, the rediscovery rate of LRCAGE reduced to 85% for transcripts that were >5 kb, whereas LRhex's rediscovery rate was consistently at ∼95% for transcripts of all length (Supplemental Fig. S4B).

### Long-read CAGE improves recall for TSSs located within regions of low mappability scores

As increased read length will expand the uniquely mappable portion of a genome (Derrien et al. 2012; Karimzadeh et al. 2018), we next examined whether long reads could improve detection of active GTSSs with low mappability scores. In line with previous studies, we observed the increased recall by long-read CAGE for active, low mappability GTSSs (Fig. 1D; Supplemental Table S2). Although the recalls were comparable at ∼80% for highly mappable GTSSs, long-read CAGE had a recall of 51% for less mappable GTSSs, whereas nanoCAGE had a recall of 34% using 2 × 75 bp and 40% using 2 × 150 bp. Moreover, 5% and 4% of active GTSSs uniquely detected by LRCAGE and LRhex had low mappability scores compared with 2% and 1% of active GTSSs uniquely detected by nanoCAGE (Supplemental Fig. S5A–C). To further support the accuracy of active GTSS detection, we examined chromatin accessibility of active GTSSs using ATAC-seq data from the same cell line. As expected, ∼99% active GTSSs detected by both long-read CAGE and nanoCAGE were in ATAC-seq peaks when mappability scores of the corresponding regions were high (Supplemental Fig. S6A,C). Active GTSSs uniquely detected by long-read CAGE or nanoCAGE also had ∼95% overlap with ATAC-seq peaks. In contrast, for active GTSSs located in regions with low mappability scores, ∼74% of active GTSSs detected by both long-read CAGE and nanoCAGE overlapped ATAC peaks, whereas ∼54% of LRhex-unique GTSSs and 22% nanoCAGE-unique GTSSs were in ATAC-seq peaks, respectively (Supplemental Fig. S6B). Percentages of ATAC peak overlap for LRCAGE-unique and nanoCAGE-unique GTSSs were 47% and 42%, respectively (Supplemental Fig. S6D). Although it is important to note that the ATAC-seq analysis was not immune to biases caused by mappability, these results provide additional support that long-read CAGE could identify bona fide promoters residing in regions with low mappability better than nanoCAGE.

**Figure 2.**
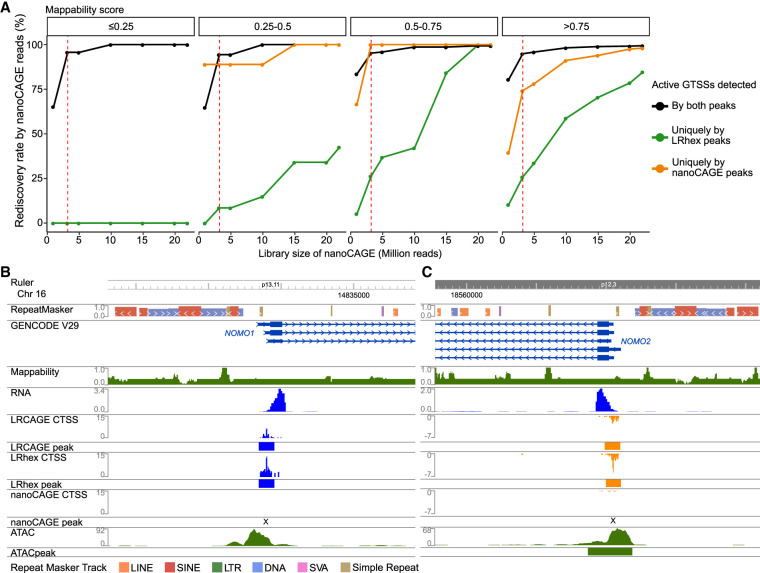
Superior recall of long-read CAGE data for low mappability regions compared to nanoCAGE. (*A*) Rediscovery rate by nanoCAGE reads as a function of sequencing depth. From the pairwise comparison of active GTSSs detected by nanoCAGE and LRhex peaks from 3 million reads (Fig. 1B), active GTSSs are classified into three groups: (1) detected by both peaks, (2) uniquely detected by LRhex peaks, and (3) uniquely detected by nanoCAGE peaks. For each group, the rediscovery rate by nanoCAGE reads is the number of rediscovered active GTSSs divided by total number of active GTSSs. Active GTSSs are counted as rediscovered if having CAGE TSSs (CTSSs) with at least two nanoCAGE reads within a ±200-bp window. (*B*) Browser view of *NOMO1* GTSS. (*C*) Browser view of *NOMO2* GTSS.

Because recall is often dependent on sequencing depth, we examined whether the increased sequencing depth of short-read nanoCAGE data could compensate for its lower recall for active GTSSs with low mappability scores (Fig. 2A). From the pairwise comparison of active GTSSs detected by either nanoCAGE peaks or LRhex peaks using 3 million reads, we focused on 1378 active GTSSs uniquely detected by LRhex peaks (Fig. 1B). We reasoned that if the increased sequencing depth of the nanoCAGE library could compensate for lower recall for active GTSSs with low mappability scores, these 1378 active GTSSs would be rediscovered by the deeply sequenced nanoCAGE library of 22 million reads, which is above the standard for short-read RNA-seq established by the ENCODE Consortium (The ENCODE Project Consortium 2020). For GTSSs with high mappability scores, >80% were detected by the deeply sequenced nanoCAGE library. However, <45% of GTSSs in low mappability scores were identified, and none of the GTSSs within ≤0.25 mappability scores were detected by the deeply sequenced nanoCAGE library (Fig. 2A). Thus, increasing the sequencing depth of nanoCAGE cannot further increase recall to detect active GTSSs with mappability scores of ≤0.25, which highlights the advantage specific to long-read technology.

Next, we calculated the rediscovery rate again using 16,992 active GTSSs detected by LRhex peaks as a benchmark. Consistent with previous results, both nanoCAGE and LRCAGE showed a similar increasing pattern as a function of expression levels, reaching 87% and 91% for active GTSSs with ≥1 TPM by nanoCAGE and LRCAGE, respectively (Supplemental Fig. S7A). Reinforcing the difference being a function of mappability, nanoCAGE had a 62% rediscovery rate for active GTSSs with ≤0.5 mappability scores, whereas LRCAGE had 92% (Supplemental Fig. S7C). As a function of transcript length, the rediscovery rates by nanoCAGE were robust at 88%, but those of LRCAGE were decreasing from 93% for GTSSs of 3- to 4-kbp-long transcripts to 85% for GTSSs of >6-kbp-long transcripts (Supplemental Fig. S7B). Figure 2, B and C, illustrates two examples of active GTSSs of two paralog protein-coding genes, *NOMO1* and *NOMO2*. Because of the 99% sequence similarity of *NOMO1*, *NOMO2*, and *NOMO3*, their GTSSs were in regions with mappability scores of ∼0.3. Both GTSSs were identified by long-read CAGE but not by nanoCAGE. Altogether, long-read CAGE has shown a superior recall for GTSSs with mappability scores of ≤0.5 compared with nanoCAGE.

### Cryptic TSSs are enriched in evolutionarily young transposable elements

We reasoned that the superior recall of long-read CAGE would enable better detection of cryptic TSSs, especially those in regions with low mappability scores. We defined cryptic TSSs as CAGE peaks at least 200 bp away from annotated GTSSs in a strand-specific manner and not overlapping GENCODE exons to further remove potential false positives from incomplete reverse transcription. We obtained 1594, 2157, and 1287 cryptic TSSs from LRCAGE, LRhex, and nanoCAGE, respectively; 63%–79% of cryptic TSSs were detected in more than one library, and 28%–45% of them were detected in all three libraries using 200 bp as the distance cutoff. Leveraging ATAC-seq data, we showed that 92%–93% of cryptic TSSs with >0.5 mappability scores and 70%–79% of cryptic TSSs with ≤0.5 mappability scores were in accessible chromatin (Supplemental Fig. S8A), supporting that these were bona fide TSSs.

**Figure 3.**
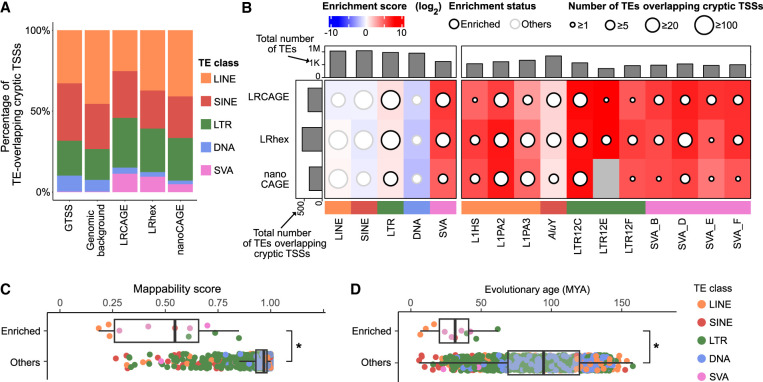
TE subfamilies enriched with cryptic TSSs have lower mappability scores and are evolutionarily younger than other TE subfamilies. (*A*) Proportion of TEs overlapping cryptic TSSs by TE class. (*B*) Cryptic TSS enrichment heatmap by TE class and TE subfamily. Enriched TE subfamilies were defined as having 1.5 or more enrichment scores, 100 or more total TE elements, and five or more TE elements overlapping cryptic TSSs. (*C*) Mappability scores of TE subfamilies by enrichment status with cryptic TSSs. (*D*) Evolutionary ages of TE subfamilies by enrichment status with cryptic TSSs. (*C*,*D*) Enriched indicates 11 TE subfamilies; others, 1075 TE subfamilies.

The human genome contains TEs of different ages and varying degrees of mappability (Sexton and Han 2019; O'Neill et al. 2020). However, 76-bp paired-end data were incapable of mapping evolutionarily young TEs (Sexton and Han 2019; O'Neill et al. 2020). Recently, Berrens et al. (2022) showed that long-read technology could quantify locus-level expression of L1 TEs in mouse genome, which are undetected by short-read technology. Therefore, we reasoned that long-read CAGE would identify cryptic TSSs from evolutionarily young TEs more effectively than nanoCAGE. Of note, the cell line used in this study, H1299, is *TP53* null, which is known to be associated with an increased number of TE-chimeric transcripts (Shah et al. 2023). Overall, 26.5%–30.4% of cryptic TSSs identified by LRCAGE, LRhex, and nanoCAGE were within TEs compared with 20% of GTSSs overlapping with TEs (Supplemental Fig. S8B). Notably, the SVA class, which had the lowest average mappability scores among TE classes, contributed higher fractions of cryptic TSSs in long-read CAGE compared with the fractions in nanoCAGE, suggesting greater sensitivity of long-read CAGE data to detect cryptic TSSs in the SVA class (Fig. 3A; Supplemental Fig. S8B,C). For example, a cryptic TSS from SVA_F with <0.1 mappability scores was detected by long-read CAGE but not by nanoCAGE (Supplemental Fig. S9). To identify sequence context for their promoter activities, we anchored CTSS signals and peaks of all SVA elements to the consensus SVA_D sequence (Supplemental Methods; Supplemental Fig. S10A). Of the 56 SVA elements having long-read CAGE or nanoCAGE peaks in the sense orientation, 92% had promoter activities in the *Alu*-like domain, which was concordant with the predicted TSSs (Hancks and Kazazian 2010). Five transcription binding sites in the *Alu*-like domain were associated with promoter activities, and their corresponding transcription factors were expressed at ≥1 TPM (tetrachoric correlation coefficient: 0.54–0.60; chi-square test: <0.01 *P*-value) (Supplemental Fig. S10B), suggesting that they might mediate SVA's promoter activities.

Next, we explored which TE subfamilies were enriched with cryptic TSSs (Supplemental Methods). Using a 1.5-fold enrichment score as a cutoff, we revealed 11 subfamilies enriched for having cryptic TSSs, including four SVA subfamilies (Fig. 3B). Compared with TE subfamilies that were not enriched with cryptic TSSs, these 11 subfamilies had lower mappability scores and younger evolutionary ages (Wilcoxon test: <0.05 *P*-value; Methods) (Fig. 3C,D). These subfamilies are specific to humans (L1HS, SVA_E, SVA_F), Hominidae (L1PA2, SVA_B, SVA_D), Hominoidea (L1PA3, LTR12C, LTR12E), Catarrhini (LTR12F), or Primates (*Alu*Y). With the exception of the *Alu*Y subfamily, we observed that >60% of TEs overlapping cryptic TSSs were in the sense orientation (Supplemental Fig. S11A,B). These prevalent sense-orientation patterns are consistent with prior knowledge: L1, SVA, and LTR elements have sense-oriented RNA Pol II promoters (Hancks and Kazazian 2010; Babaian and Mager 2016; Chuong et al. 2017). Although *Alu*Y elements have sense-oriented RNA Pol III promoters (Babaian and Mager 2016), Pol III transcripts are not polyadenylated (Sisodia et al. 1987) and thereby were depleted in our sequenced data owing to poly(A) RNA pulldown step (Supplemental Fig. S1A). Also, antisense promoters in *Alu* elements have been reported to produce poly(A) RNAs (Jang et al. 2019; Shah et al. 2023). We suspect that some relatively young TEs retain promoter activities during human evolution, but their promoter activities remain unannotated, in part owing to their low mappability scores.

In addition to TEs, we also investigated promoters in other repeat elements (REs). Compared with 16.6% of GTSSs overlapping with REs, 16.7%–18.5% of cryptic TSSs identified by LRCAGE, LRhex, and nanoCAGE were within REs (Supplemental Fig. S12A). Notably, percentages of cryptic TSSs in the satellite class were higher than that of GTSSs; 40%–50% of cryptic TSSs in the satellite class were attributed to SST1 subfamily, which has promoter/enhancer-like RNA polymerase occupancy patterns in human CHM13 cells (Hoyt et al. 2022). We did not observe any RE subfamily that contributed higher percentages to cryptic TSSs in long-read CAGE than in nanoCAGE, which could be owing to their relatively high mappability scores (Supplemental Fig. S12B).

### Newly characterized transcripts can be translated into unannotated proteins and antigens

**Figure 4.**
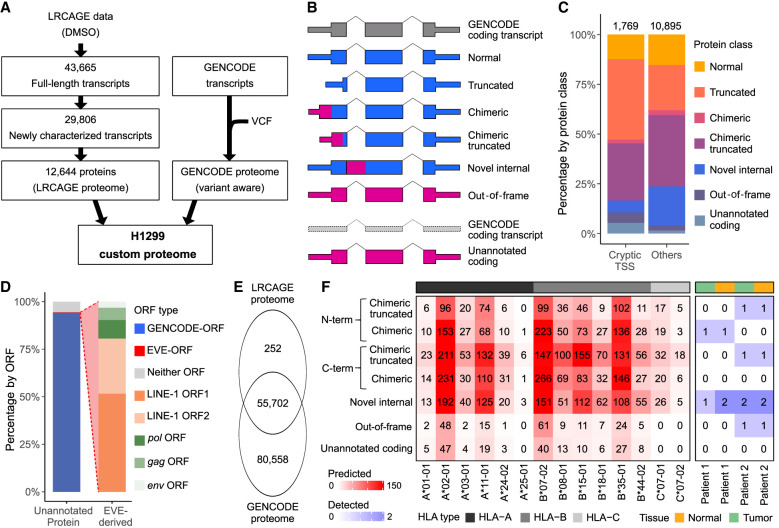
LRCAGE proteome enables the detection of unannotated proteins and noncanonical antigens in H1299 cells. (*A*) Flowchart of preparing H1299 custom proteome using LRCAGE data in H1299 cells. (*B*) Classification scheme of proteins. Pink indicates unannotated peptide sequences; blue, peptide sequences identical to GENCODE proteins. (*C*) Proportions of proteins in the LRCAGE proteome by protein class. (*D*) Percentages of unannotated proteins by their ORFs relative to GENCODE and EVE-ORFs. (GENCODE-ORF) Unannotated proteins with truncated, chimeric, chimeric truncated, or novel internal class (Fig. 1B), (EVE-ORF) unannotated proteins with out-of-frame or unannotated coding class and overlapping EVE-ORFs; and (neither ORF) unannotated proteins overlapping neither ORF type. (*E*) Venn diagram of peptides in H1299 whole-cell lysate MS data by proteome databases. (*F*) Heatmap of noncanonical antigens predicted for 14 HLA alleles (*left*) and antigens from H1299 HLA-pulldown LC-MS/MS data of two lung cancer patients (*right*). C*04:01 has no antigens predicted and was omitted.

In addition to discovering TSSs, long-read sequencing provides a strong advantage in detecting transcript isoform diversity in human and mouse samples (Tilgner et al. 2015; Leung et al. 2021; Glinos et al. 2022). To investigate full-length transcripts, we used LRCAGE reads to call transcripts because LRCAGE reads span from the 5′ end to poly(A) tail. Then, we filtered out transcripts whose 5′ ends were not supported by the merged peaks of LRCAGE and nanoCAGE peaks, resulting in 43,665 full-length transcripts (Fig. 4A; Supplemental Fig. S13A). These included 594 transcripts from 229 GENCODE pseudogenes, of which 10% (23) were also reported in a previous study using PacBio Iso-Seq data (Troskie et al. 2021; Supplemental Fig. S13B,C). Based on transcript classification using TALON (Wyman et al. 2020), 29,806 of these transcripts were previously uncharacterized (Supplemental Fig. S13D; Supplemental Table S3), including 4910 from cryptic TSSs. Ninety-three percent of the newly characterized transcripts are multiexon transcripts. Newly characterized transcripts were shorter on average (cryptic TSS-derived transcripts: 1830 bp; GTSS-derived transcripts: 1884 bp) than GENCODE coding transcripts (2237 bp) but were longer than lincRNA transcripts (691 bp) (Supplemental Fig. S13E). As a positive control, the onco-exapted transcript of *LIN28B* derived from *Alu*Jb was readily detected (Supplemental Fig. S14; Jang et al. 2019). Twenty-eight percent (8448) of the newly characterized transcripts contain exonic sequences from 10,426 TEs, including 7628 TEs in which their entire sequences contribute to exons. We also observed that cryptic inner exons overlapping TEs are enriched in *Alu* subfamilies in antisense orientation (Supplemental Fig. S13F–H), concordant with previous studies (Zarnack et al. 2013; Attig et al. 2016).

We explored the hypothesis that newly characterized long-read CAGE transcripts could be translated to provide additional antigens. To identify potential novel proteins, we generated a H1299 custom proteome by combining a LRCAGE proteome with a variant-aware GENCODE proteome (Fig. 4A). We prepared the LRCAGE proteome by in silico translation of newly characterized transcripts (Methods). Of 12,664 proteins encoded by these transcripts, 10,774 were unannotated proteins, which had the potential to produce noncanonical antigens (Fig. 4B,C). For ORFs, 94% of unannotated proteins joined with GENCODE-ORFs, <1% joined with endogenous viral element (EVE)-ORFs, and the remaining 6% joined neither ORF type (Fig. 4D). Among EVE-ORFs, LINE-1 ORF1 and ORF2 contributed the most followed by *pol*, *gag*, and *env* (Fig. 4D). Of the 25 proteins joining LINE-1 ORFs, 68% (17) and 48% (12) were encoded by L1- and L1PA2-derived transcripts, respectively (Supplemental Table S4).

In H1299 whole-cell lysate LC-MS/MS data (Choi et al. 2020), 252 peptides were uniquely identified with the LRCAGE proteome (Fig. 4E; Supplemental Table S5). Peptides uniquely encoded by cryptic TSSs were enriched in out-of-frame or unannotated coding proteins compared with others (Supplemental Fig. S15A). For example, TALONG000051076_NP_1 was encoded by a cryptic TSS-derived transcript located within an L1HS element in the intron of the *EMBP1* gene (Supplemental Fig. S15B). Using H1299 whole-cell lysate LC-MS/MS data, we identified 10 peptides aligned to this protein but only one peptide uniquely aligned to TALONG000051076_NP_1 owing to its sequence similarity to LINE-1 ORF1 (Supplemental Fig. S15C). We found another two peptides aligned to LINE-1 ORF1 but not derived from TALONG000051076_NP_1. We also detected a peptide, DVDRYQAVLANLLLEEDNK, that aligned to the chimeric region of a protein, ENSG0000084070.11_NP_1, from a cryptic TSS-derived *SMAP2* isoform (Supplemental Fig. S15D). Additionally, we screened whole-cell lysate LC-MS3 data of CPTAC LUAD cohorts (Gillette et al. 2020) and identified 557 peptides unique to the LRCAGE proteome. These results highlight that the usage of LRCAGE in building the custom proteome database enables the identification of novel proteins not only in H1299 cells but also in CPTAC LUAD samples.

Next, we investigated antigens that were uniquely detected by the LRCAGE proteome. First, using antigens predicted by NetMHC (Andreatta and Nielsen 2016), we found 11 out of 14 prevalent HLA alleles had at least 100 putative antigens unique to the LRCAGE proteome (9-mer) (Fig. 4F). Also, from the HLA-pulldown LC-MS/MS data of two lung cancer patients, we identified eight antigens unique to the LRCAGE proteome (Fig. 4F; Supplemental Table S6). For instance, the TPYRKQQSL antigen was derived from the 5′ end chimeric truncated region of an unannotated protein, ENSG00000153250.19_NP_3, and was detected in patient 2 samples (Supplemental Fig. S16A). Another peptide, ILAQEIVKV was observed in the 3′ end chimeric truncated region of an unannotated protein, ENSG00000142230.11_NP_1, in patient 2 samples (Supplemental Fig. S16B). Together, our findings substantiate the use of a LRCAGE proteome to detect noncanonical antigens from unannotated proteins.

### LRCAGE identifies TE transcripts induced upon epigenetic treatment

Previous studies reported that epigenetic treatment induces TE transcripts, which may encode novel proteins and antigens (Brocks et al. 2017; Jang et al. 2019; Jones et al. 2019; Kong et al. 2019). To investigate the impact of epigenetic treatment using the LRCAGE proteome, we profiled promoter activities and their transcripts from H1299 cells treated with epigenetic drugs. We used the combinatory treatment (DACSB; Methods) by combining DNMT inhibitor (decitabine) and HDAC inhibitor (SB939). This epigenetic treatment regime has been shown to maximize the transcriptional reactivation of cryptic promoters (Brocks et al. 2017).

Combining LRCAGE and nanoCAGE data, we identified 26,859 consensus peaks and 868 up-regulated peaks upon epigenetic drug treatment (fourfold or more, <0.05 adj. *P*-value; Methods). Seventy-four percent (644) of the up-regulated peaks overlapped LTR elements (Supplemental Fig. S17A), and 76% (658) were cryptic TSSs. We confirmed higher peak intensity of LRCAGE over nanoCAGE in peaks with low mappability scores, as expected (Wilcoxon test, *P* < 0.05) (Supplemental Fig. S17B). Higher sensitivity in lowly mappable regions was critical to detecting up-regulated cryptic TSSs because these peaks were enriched in repetitive sequences, including young TEs.

**Figure 5.**
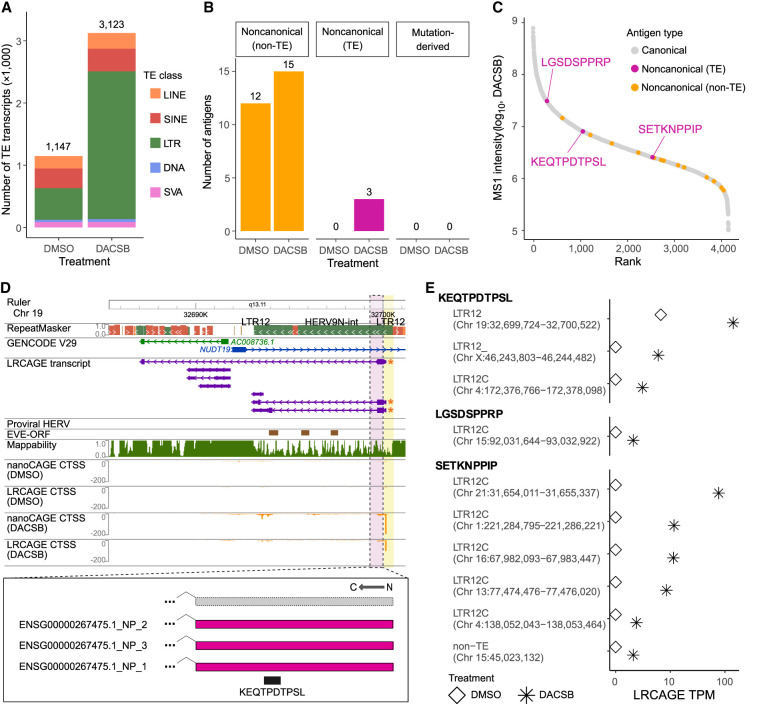
LRCAGE proteome identifies epigenetic drug-induced TE antigens in H1299. (*A*) Newly characterized TE transcripts as a function of epigenetic treatment annotated with TE class at 5′ end. (*B*) Number of noncanonical antigens as a function of epigenetic treatment in H1299 cells annotated with antigen type. (*C*) MS1 intensity of antigens in H1299 cells. (*D*) Browser view of LTR12 transcripts encoding a drug-induced TE antigen, KEQTPDTPSL. A TSS is marked with a yellow bar, and a protein-coding region is marked with a pink bar. Newly characterized transcripts encoding a noncanonical antigen are marked with a red asterisk (*). (*E*) Expression level of TE loci encoding drug-induced TE antigens.

To understand the impact of epigenetic treatment to TE transcripts, we used LRCAGE data and identified the full-length structure of 48,153 newly characterized transcripts, including 3244 TE transcripts (Supplemental Table S7). Epigenetic therapy increased the number of TE transcripts by 2.7-fold but not that of non-TE transcripts (Fig. 5A; Supplemental Fig. S18A). This was primarily by activation of LTR-derived promoters (Supplemental Fig. S18B). Because LTRs could function as promoters of GENCODE genes, proviral human endogenous retrovirus (HERV) or unannotated genes, we classified LTR-derived transcripts (LTR transcripts) by their overlap with gene and proviral HERV annotations. Upon epigenetic treatment, the mean expression levels of multiexon LTR transcripts increased by more than sevenfold (Supplemental Fig. S18C). Using 1 TPM as the expression-level cutoff, the number of multiexon LTR transcripts increased by 1099; 569, 464, and 66 overlapped unannotated, genic, and proviral HERV regions, respectively (Supplemental Fig. S18D). These transcripts were transcribed from 530 LTRs, of which 63% expressed a single transcript per LTR (Supplemental Fig. S18E). These results suggest that the drug-induced increase of TE transcripts is mostly from LTR elements and that LTR transcripts from unannotated regions contribute the most to the drug-induced increase followed by those from genic and proviral HERV regions.

### LRCAGE-identified transcripts produce drug-induced TE antigens in lung cancer and glioblastoma cells

To explore whether the drug-induced novel TE transcripts could provide an additional source of potential antigens, we prepared a H1299 custom proteome using LRCAGE data of H1299 cells treated with DACSB (Supplemental Fig. S19A) and generated HLA-pulldown LC-MS/MS data. Using the H1299 custom proteome, we identified 4623 antigens, including 20 noncanonical antigens and three noncanonical TE antigens (Fig. 5B). We confirmed the purity of antigens using size distribution (Supplemental Fig. S19B). The sensitivity of detecting antigens predicted by NetMHCpan (9-mer, 500 nM) increased as a function of LRCAGE expression levels (Supplemental Fig. S19C), as did sensitivity of detecting in silico digested peptides (Supplemental Fig. S19D). Overall, these suggested that most antigens were bona fide antigens, but their detection sensitivity was limited by the abundance of antigens in samples. Next, we evaluated the identification accuracy of noncanonical antigens by examining a Spearman's correlation of the observed and predicted peptide retention time in the liquid chromatography (Chong et al. 2020; Cuevas et al. 2021). After excluding one outlier in noncanonical antigens, the retention time correlation for noncanonical antigens (0.93) was comparable to that of canonical antigens (0.93), supporting that noncanonical antigens were accurately identified (Supplemental Fig. S19E).

In line with previous studies, epigenetic therapy increased the number of antigens from 3455 to 4166 (Shraibman et al. 2016; Kong et al. 2019), including the 12 and 18 noncanonical antigens, respectively. Although none of the mutation-derived antigens were detected, we detected three noncanonical antigens from drug-induced TE transcripts (drug-induced TE antigens) (Fig. 5B; Supplemental Table S8): KEQTPDTPSL, LGSDSPPRP, and SETKNPPIP. Drug-induced TE antigens were defined as antigens uniquely detected under DACSB treatment with >90% expression from TE-transcripts and more than 10-fold expression-level changes compared with DMSO treatment (Fig. 5D,E). Of these antigens, two were encoded by LTR elements. The relative abundance based on MS1 intensity indicated that KEQTPDTPSL and LGSDSPPRP were within the top 25% and SETKNPPIP was within the top 61% in H1299 cells treated with DACSB (Fig. 5C). Next, to assess the therapeutic potential of drug-induced TE antigens as a cancer vaccine, we predicted immunogenic scores based on antigen presentation and TCR recognition using PRIME (Schmidt et al. 2021). Two antigens, KEQTPDTPSL and SETKNPPIP, were predicted to be immunogenic in the top 1% compared with a background of randomly selected peptides from the human proteome. Taken together, these analyses show that drug-induced TE antigens expand the pool of targetable antigens for immunotherapy.

Finally, we asked whether LRCAGE could help detect drug-induced TE antigens in other cancer types. Using our H1299 custom proteome, we examined the HLA-pulldown LC-MS/MS data of three glioblastoma cell lines treated with decitabine (DAC) (Shraibman et al. 2016), and identified 14,228–18,021 antigens for each glioblastoma cell line, including 98–101 noncanonical antigens and seven to nine noncanonical TE antigens. Using expression levels based on H1299 LRCAGE data, we detected three drug-induced TE antigens (Supplemental Fig. S20A,B). One antigen, ILDFQPPEL, was shared across all three glioblastoma cell lines with varying relative abundance and was predicted to be immunogenic in all three cell lines (Supplemental Fig. S20C–E). This antigen was produced from a MLT1K promoter, which was also activated in H1299 cells upon DACSB treatment (Supplemental Fig. S20F), indicating that the peptide was not detected, possibly owing to low abundance. Altogether, these analyses suggest that LRCAGE enables detecting drug-induced TE antigens in both on-target and off-target cancer cell types.

### LRCAGE identifies a HERV9 locus producing an *env*-derived antigen upon epigenetic treatment

Previous studies reported that epigenetic treatment up-regulates the expression of ERVs using a subfamily expression-level analysis based on short-read RNA-seq or CAGE-seq (Brocks et al. 2017; Daskalakis et al. 2018; McDonald et al. 2021). Using LRCAGE data, we quantified locus-level expression changes of proviral HERVs upon epigenetic treatment. In H1299 cells treated with DACSB, we detected 89 newly characterized multiexon transcripts from 46 proviral HERV loci (HERV transcripts) (Supplemental Table S9). Most transcripts (71 from 43 loci) were up-regulated by fourfold or more upon epigenetic treatment, and 67 of these up-regulated transcripts were exclusively expressed upon DACSB treatment (Supplemental Fig. S21A). Compared with all the proviral HERV clade expressing drug-induced transcripts, the proviral HERV9 clade, which consists of HERV9-int as an internal sequence with flanking LTR12 elements, was enriched in up-regulated transcripts (enrichment score: 20.8) (Supplemental Methods). This observation is concordant with previous studies using short-read RNA-seq or CAGE-seq (Brocks et al. 2017; Daskalakis et al. 2018; McDonald et al. 2021).

In addition, a recent study reported that conserved EVEs can produce antigens in glioblastoma cells (Bonté et al. 2022). EVE is a host genome DNA sequence that originated from ancient viral infection of germ cells (Nakagawa and Takahashi 2016), and open reading frames of EVEs (EVE-ORFs) can encode proteins (Pastuzyn et al. 2018; Ueda et al. 2020). Across 46 transcriptionally-active proviral HERV loci in H1299 cells, only 39% of loci express transcripts containing EVE-ORFs, presumably owing to splicing. This implies that not all EVE-ORFs within active proviral HERV loci have the potential to produce antigens. To find all potential antigens from HERV transcripts, we expanded the proteome of HERV transcripts by using ≥25-amino-acid-long peptides from three-frame translation, amending our previous method using the longest peptide with 100 or more amino acids. This change increased the number of possible coding HERV transcripts from six to 87. Using the expanded proteome, we identified one *env*-derived antigen, PAGTFTGLE, from a HERV9 locus under the DACSB treatment condition (Supplemental Fig. S21B). The expression levels of this antigen increased from 0.0 TPM to 14.0 TPM. Based on MS1 intensity, the relative abundance of this antigen was in the top 35% (Supplemental Fig. S21C), but this antigen was not predicted to be immunogenic (percent rank by immunogenic score rank: top 5.6%). Altogether, these analyses show that LRCAGE enables the identification of precise proviral HERV loci, which produce EVE-ORF-derived antigens.

## Discussion

To detect putative antigens in HLA-pulldown LC-MS/MS data, current immunopeptidome analysis often identifies peptides using reference proteome databases (Purcell et al. 2019; Chong et al. 2020). Several groups have successfully discovered noncanonical peptides using proteome databases augmented by adding assembled transcripts based on short-read RNA-seq (Laumont et al. 2016; Attig et al. 2019; Chong et al. 2020; Cuevas et al. 2021). However, transcript assembly using short-read RNA-seq, particularly for TE transcripts, has two limitations. First, short reads from repetitive regions are discarded (Lee and Schatz 2012; Conesa et al. 2016; Sexton and Han 2019). Second, transcript assembly is highly dependent on read length and read coverage of the transcript (Steijger et al. 2013; Boley et al. 2014; Pertea et al. 2015). To tackle these challenges, we developed computational toolkits for long-read CAGE data, LRCAGE and LRhex, to profile cryptic TSSs, uncharacterized transcripts, and unannotated proteins for immunopeptidome analysis.

We explored whether LRCAGE improves identification of noncanonical antigens from previously uncharacterized transcripts, including those from cryptic TSSs. Using H1299 cells, we first showed greater sensitivity of long-read CAGE data over short-read nanoCAGE for detecting TSSs within low mappable regions. Next, using LRCAGE data, we profiled 29,806 previously uncharacterized transcripts, including 4910 from cryptic TSSs. Ultimately, to detect noncanonical antigens, we built a custom proteome database augmented with newly characterized transcripts. From two lung cancer patients, we identified eight noncanonical antigens. These findings emphasize that a neglected source of antigens can be found using LRCAGE data. Furthermore, using a H1299 custom proteome database augmented by LRCAGE data, we could detect noncanonical antigens from reactivation of TEs in H1299 and three glioblastoma cell lines. In H1299 cells, although we could not verify any mutation-derived antigens, we could detect three drug-induced TE antigens, suggesting that drug-induced TE antigens can serve as a pool of antigens even when mutation-derived antigens are rare or nonexistent. Of the three drug-induced TE antigens in glioblastoma cells, one antigen was shared and predicted to be immunogenic across all three cell lines, further supporting the idea that drug-inducible antigens may be attractive targets for immunotherapy. To detect noncanonical antigens in HLA-pulldown LC-MS/MS data, it is critical to expand the reference proteome database by using LRCAGE data.

A recent study has reported that EVE-ORFs can produce antigens based on short-read RNA-seq data (Bonté et al. 2022). However, because of multimapping issues, it remains unknown which proviral HERV loci are producing EVE-ORF-derived antigens. Leveraging LRCAGE data, we could pinpoint the specific proviral HERV loci that were expressed upon epigenetic treatment and quantify their locus-level expression-level changes, which is not possible using short-read RNA-seq or CAGE-seq data. Notably, we found that most transcriptionally-active proviral HERV loci do not have EVE-ORFs in their transcripts, precluding the potential of EVE-ORFs to produce antigens. Nonetheless, we detected one locus encoding a drug-induced *env*-derived antigen. These findings emphasize the importance of using LRCAGE data to better understand proviral HERV transcriptional activity and their subsequent ERV-ORF-derived antigens.

One caveat of our approach is a reduced sensitivity for transcripts with low expression levels. Using a LRCAGE library of 3 million reads, 42.5% of active GTSSs with ≤0.5 TPM were detected by LRCAGE reads compared with 76.8% of GTSSs with >3 TPM. However, it is known that sensitivity increases with high sequencing depth (Wyman et al. 2020; Gao et al. 2023), so we anticipate that this limitation will be mitigated. For instance, from 2020 to 2023, sequencing cost for 30× human genome has dropped from $5805 to $995 for PacBio and from $2835 to $600 for ONT, indicating sequencing throughput per dollar has increased by more than fourfold (Logsdon et al. 2020; Kovaka et al. 2023). In addition, new library preparation techniques such as concatenating cDNAs before sequencing (Al'Khafaji et al. 2023) can further push long-read sequencing depth closer to that of short reads. Another limitation of LRCAGE data is a bias against >5-kbp-long transcripts, probably owing to incomplete reverse transcription. To overcome this limitation, size-selection of cDNAs before sequencing could be a potential solution. This has been successfully shown to sequence a 106-kbp-long transcript from the mouse *Ttn* gene (Uapinyoying et al. 2020).

In conclusion, we developed a genomics toolkit for long-read CAGE data to investigate promoters in regions with low mappability scores, previously uncharacterized transcripts, and antigens. Our work paves the way for further investigation into the role of TE transcription in the immunopeptidomes across tissues or in a developmental stage-specific manner or upon extrinsic cues or in diseases including cancer and autoimmunity. With increased throughput of long-read sequencing platforms, we expect our toolkits will further advance our knowledge of lowly expressed TE transcripts and their contribution to immunopeptidome.

## Methods

### Sample preparation

H1299 cells were cultured in RPMI-1640 supplemented with 10% FBS. For epigenetic treatment, H1299 cells were treated with 100 nM decitabine (Millipore Sigma) for 72 h and 500 nM SB939 (Cayman Chemical) for the last 18 h. The medium was changed every 24 h. Cells were harvested by trypsin dissociation for long-read CAGE and nanoCAGE library preparation and by scraping for HLA-I pulldown experiment.

### Library preparation of LRCAGE, LRhex, and nanoCAGE

To enrich our RNA samples for reverse transcription with full-length mRNA, we added two additional steps, poly(A) RNA pulldown and exonuclease treatment, compared with the conventional PacBio Iso-Seq protocol (Iso-Seq express Template Preparation for sequel I and sequel II systems). Poly(A) RNA was isolated from 2 million H1299 cells using a Dynabeads mRNA purification kit (Thermo Fisher Scientific). One hundred fifty nanograms of poly(A) RNAs was used as input for nanoCAGE, LRCAGE, and LRhex. Poly(A) RNAs were treated with Terminator 5′-phosphate-dependent exonuclease (Lucigen) to digest RNAs damaged at 5′ ends. For nanoCAGE library preparation, we followed the standard protocol (Poulain et al. 2017). For long-read CAGE library preparation, we adapted the Iso-Seq library preparation to use nanoCAGE TSO and to use poly(dT) RT primer and random hexamer RT primer for LRCAGE and LRhex, respectively (Supplemental Fig. S1A).

### Data processing for LRCAGE and LRhex

CCS reads were processed with lima (version 1.11.0; https://github.com/PacificBiosciences/barcoding), and isoseq3 refine (v3.1.2; https://github.com/PacificBiosciences/IsoSeq) to trim PCR primers and discard concatemers. The custom script was used to trim TSO and to trim 15 A's only for LRCAGE. Trimmed reads ≥250 bp long were considered full-length nonchimeric (FLNC) reads. The reference genome was prepared from the hg38 assembly by discarding alternate contigs and hardmasking pseudoautosomal regions of the Y Chromosome to avoid false-positive multimapping (https://lh3.github.io/2017/11/13/which-human-reference-genome-to-use). FLNC reads were aligned to the reference genome using minimap2 (Li 2018) with junc-bed input with the option “‐‐secondary = yes -C5 ‐‐MD -uf -G 589824 -N 20.” We used a custom script to realign to reduce spurious alignment at splice junctions. Primary, nonsupplemental, and uniquely mapped reads were used for the downstream analysis. For deduplication, we used a custom script to deduplicate by coordinates and UMIs. For LRCAGE, we filtered out reads with more than three soft-clips at either end of the read. For LRhex, we discarded aligned reads with either more than three soft-clips at the 5′ end or more than 20 soft-clips at the 3′ end.

### Active GTSSs and peak calling

Using the custom script, we extracted CTSSs from BAM files of LRCAGE, LRhex, and nanoCAGE. We called peaks using CAGEr (Haberle et al. 2015) with the paraclu algorithm (Frith et al. 2008). To reduce false-positive peaks, we used a custom transcript adapted from Cap-Filter (Cumbie et al. 2015) with 0.35 as the cutoff. We used DESeq2 to find differentially expressed peaks (Love et al. 2014). Genomic annotation of peaks was assigned using the following order: promoter, 5′ UTR, first exon, 3′ UTR, other exons, intron, and intergenic.

For benchmark analysis, 20,949 active GTSSs were defined as TSSs of GENCODE transcripts satisfying (1) from protein-coding genes, (2) with a transcript length of 0.3–1 kbp, and (3) with ≥1 TPM by both RSEM (v.1.3.1) (Li and Dewey 2011) and Salmon (v.1.3.0) (Patro et al. 2017); 32,435 active GTSSs (relaxed) were defined using the cutoff of ≥0.1 TPM by RSEM. Precision was defined as a fraction of peaks that did not overlap GTSSs. Recall was defined as a fraction of active GTSS overlapped peaks. Both recall and precision were measured using a 200-bp tolerance window of overlap.

### Evolutionary age estimation of TEs

We used the evolutionary ages for TEs directly from Choudhary et al. (2020). Briefly, the evolutionary ages of each TE were estimated by dividing the percentage divergence of each TE by neutral substitution rate, 2.2 × 10^−9^ mutations/year. To calculate the percentage divergence of each TE, the number of substitutions in each TE against its consensus sequence was divided by the length of genomic TE minus the number of insertions.

### Profiling newly characterized transcripts

TranscriptClean (Wyman and Mortazavi 2019) was applied to the LRCAGE and LRhex BAM file with the SNV VCF file as input. Error-corrected reads were classified, collapsed, and quantified by TALON (Wyman et al. 2020) using GENCODE basic annotation (v.29). TALON database outputs candidate transcripts as GTF file. We used the custom script using TALON output and a peak file to retain a list of transcripts with complete 5′ ends. Newly characterized transcripts were defined by a TALON class other than “known.” Drug-induced transcripts are defined as transcripts with ≥1 TPM in the DACSB-treated condition and 0 TPM in the DMSO-treated condition.

### Creating a proteome database for newly characterized transcripts

To build a LRCAGE proteome, we used a custom script to predict proteins from newly characterized transcripts using ANGEL (https://github.com/PacificBiosciences/ANGEL). For each transcript, the longest protein products with 100 or more amino acids were retained. Then, we filtered out proteins with NMD features (Hu et al. 2017). To prepare an H1299 custom proteome database, we combined an LRCAGE proteome with a GENCODE proteome (variant-aware) from customProDBJ (Wen et al. 2020) with a VCF file of H1299.

### HLA-I antigen analysis using HLA-pulldown LC-MS/MS data

Using a H1299 custom proteome database, raw files were analyzed with MaxQuant (v.1.6.17.0) (Tyanova et al. 2016; Cox et al. 2020) using the following setting: {type: “Standard”; digestion mode: “Unspecific”; max.peptide.length:15; peptide FDR: 5%; protein FDR 100%}. Peptides that were from potential contaminants or reverse sequences were removed. Canonical antigens were defined as antigens that align to GENCODE proteome with 100% identity by BLAT (Kent 2002). Otherwise, they were considered noncanonical antigens. Antigens encoded by TE proteins were defined as noncanonical TE antigens, and the rest were considered noncanonical non-TE antigens. For immunogenicity prediction, PRIME (Schmidt et al. 2021) was used with top 1% rank as the cutoff for being immunogenic. Peptide retention time of antigens was predicted using DeepLC (Bouwmeester et al. 2021).

## Data access

All raw sequencing data generated in this study have been submitted to the NCBI BioProject database (https://www.ncbi.nlm.nih.gov/bioproject/) under accession number PRJNA936447. The HLA-I MS data of H1299 cells generated in this study have been submitted to the ProteomeXchange (https://www.proteomexchange.org/) under data set identifier PXD040265. All custom scripts are available at GitHub (https://github.com/twlab/LRCAGE) and as Supplemental Code. The WashU Epigenome browser links for CTSS analysis and for Epigenetic treatment-induced transcripts analysis are available at https://epigenomegateway.wustl.edu/browser/?genome=hg38&sessionFile=https://wangftp.wustl.edu/~jmaeng/publication/H1299_LRCAGE_CTSS_DMSO.json and https://epigenomegateway.wustl.edu/browser/?genome=hg38&sessionFile=https://wangftp.wustl.edu/~jmaeng/publication/H1299_LRCAGE_transcripts_DACSB.json, respectively.

## Supplementary Material

Supplement 1

Supplement 2

Supplement 3

Supplement 4
