# Supplementary material for: Using long-read CAGE sequencing to profile cryptic-promoter-derived transcripts and their contribution to the immunopeptidome
Source: Genome Res. 2023 Dec;33(12):2143–55. doi: 10.1101/gr.277061.122 (PMC10760525; doi:10.1101/gr.277061.122)
Supplement: Supplement 2 [file Supplemental_Figures.pdf]

Table of Contents

Supplemental Figure S1. Scheme of long-read CAGE library preparation and browser view.

Supplemental Figure S2. Characteristics of long-read CAGE and nanoCAGE data.

Supplemental Figure S3. Gene expression correlations between LRCAGE, LRhex, nanoCAGE and RNA-seq data.

Supplemental Figure S4. Re-discovery rate against active GTSSs detected by nanoCAGE peaks as a function of expression levels, transcript length, and mappability scores.

Supplemental Figure S5. Pairwise comparison of active GTSSs detected by LRhex, LRCAGE, and nanoCAGE peaks annotated with GTSSs with low mappability scores.

Supplemental Figure S6. Percentages of active GTSSs detected by LRhex, LRCAGE, and nanoCAGE peaks supported by ATAC peaks.

Supplemental Figure S7. Re-discovery rate against active GTSSs detected by LRhex peaks as a function of expression levels, transcript length, and mappability scores.

Supplemental Figure S8. Cryptic TSSs by their overlap with ATAC peaks and TEs.

Supplemental Figure S9. Browser view of a SVA_F cryptic promoter (Chr 20:32,175,371-32,176,478) with LRCAGE and LRhex reads.

Supplemental Figure S10. Sequence context of promoter activities in SVA elements.

Supplemental Figure S11. Relative orientation of TEs for overlapping cryptic TSSs.

Supplemental Figure S12. Cryptic TSSs by their overlap with REs.

Supplemental Figure S13. Characteristics of transcripts profiled by LRCAGE data.

Supplemental Figure S14. *Alu*Jb-*LIN28B* transcripts detected by long-read CAGE data.

Supplemental Figure S15. Peptides from unannotated proteins in the LRCAGE proteome in H1299 whole cell lysate LC-MS/MS data.

Supplemental Figure S16. Browser views of noncanonical antigens of two lung cancer patients.

Supplemental Figure S17. Characteristics of consensus peaks.

Supplemental Figure S18. Increased expression of TE-derived transcripts upon epigenetic treatment.

Supplemental Figure S19. Quality assessment of antigens from HLA-pulldown LC-MS/MS data.

Supplemental Figure S20. Drug-induced noncanonical TE antigens in three glioblastoma cell lines.

Supplemental Figure S21. A proviral HERV9 locus encoding *env*-derived antigen upon epigenetic treatment.

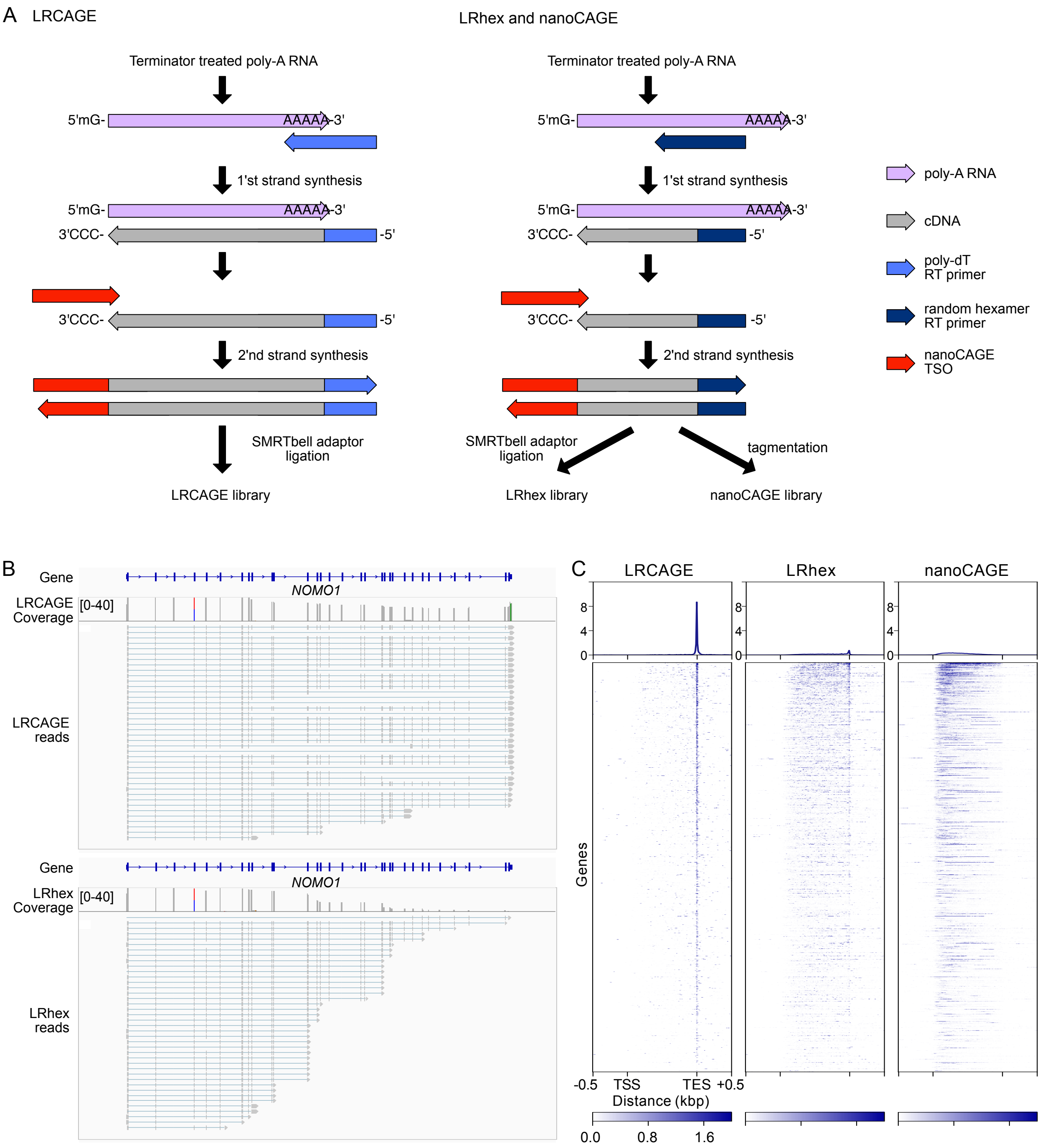

Supplemental Figure S1. Scheme of long-read CAGE library preparation and browser view. (A) LRCAGE, LRhex and nanoCAGE library preparation workflow. (B) Browser view of LRCAGE and LRhex reads at *NOMO1* gene. For visualization, 40 reads starting from *NOMO1* GTSS are randomly selected from 142 LRCAGE reads and 170 LRhex reads. (C) Heatmap of coverage

across gene bodies. For coverage, 3' ends of read were used for LRCAGE and LRhex. For paired-end nanoCAGE, 5' ends of read 2 were used.

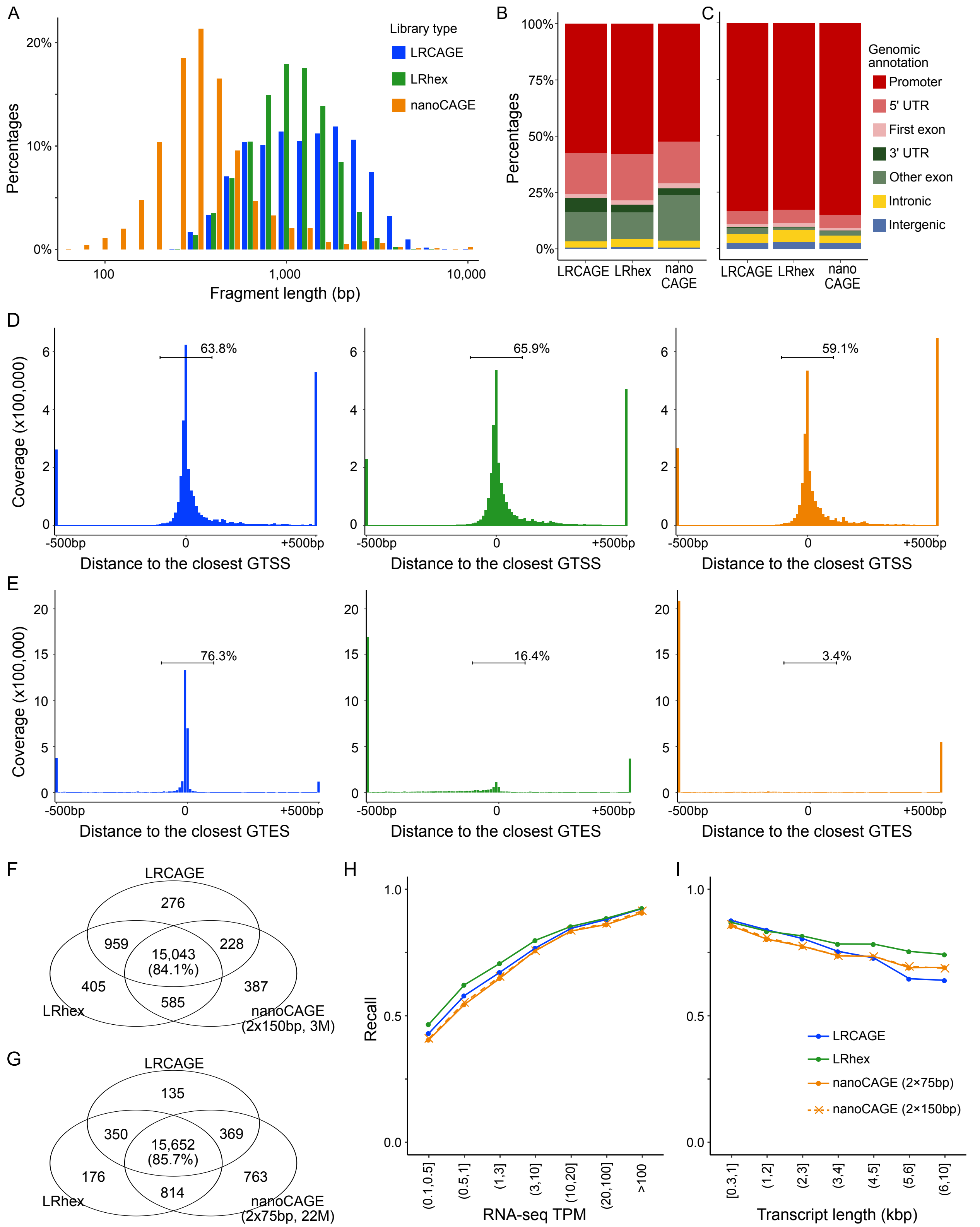

Supplemental Figure S2. Characteristics of long-read CAGE and nanoCAGE data. (A) Fragment size distribution. (B-C) Genomic annotation of 5' ends of LRCAGE, LRhex, and nanoCAGE reads

(B) and peaks (C). (D) Coverage at the closest GTSSs by 5' ends of LRCAGE, LRhex and nanoCAGE reads. (E) Coverage at the closest GTESs by 3' ends of LRCAGE and LRhex reads, and by 5' ends of read 2 for nanoCAGE. (F) Venn diagram showing intersections of active GTSSs detected by LRCAGE, LRhex and nanoCAGE (2×150bp) peaks using 3 million reads. (G) Venn diagram showing intersections of active GTSSs detected by LRCAGE and LRhex peaks using 3 million reads and nanoCAGE (2×75bp) peaks using 22 million reads. (H-I) Recall by LRCAGE, LRhex, and nanoCAGE as a function of RNA-seq expression levels (H), and transcript length (I).

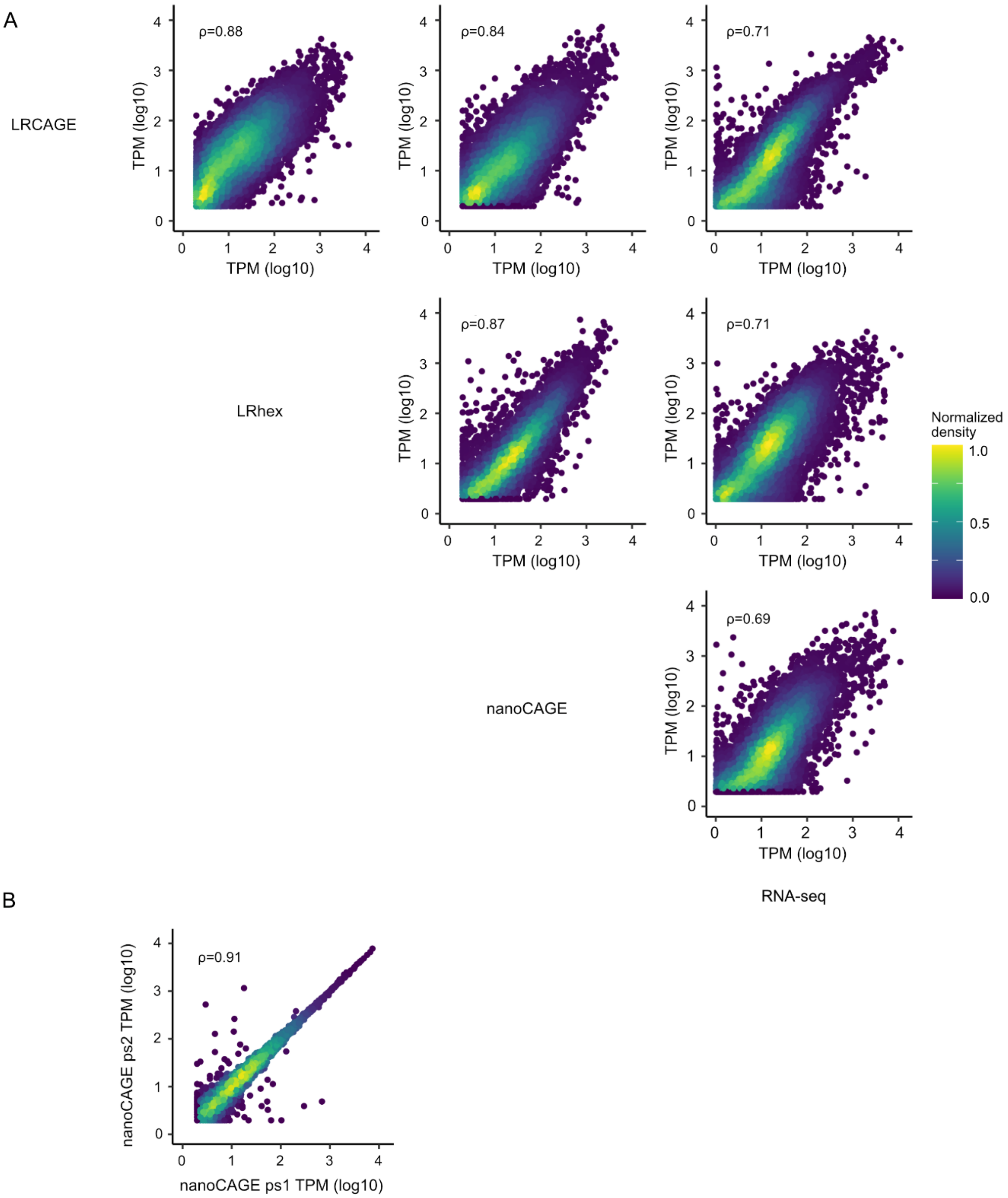

Supplemental Figure S3 Gene expression correlations between LRCAGE, LRhex, nanoCAGE and RNA-seq data. (A) Pairwise scatterplots of gene expression levels based on RNA-seq and peak strengths of LRCAGE, LRhex, nanoCAGE peaks. (B) Scatterplot of gene expression levels based on peak strengths of nanoCAGE pseudo-replicates.

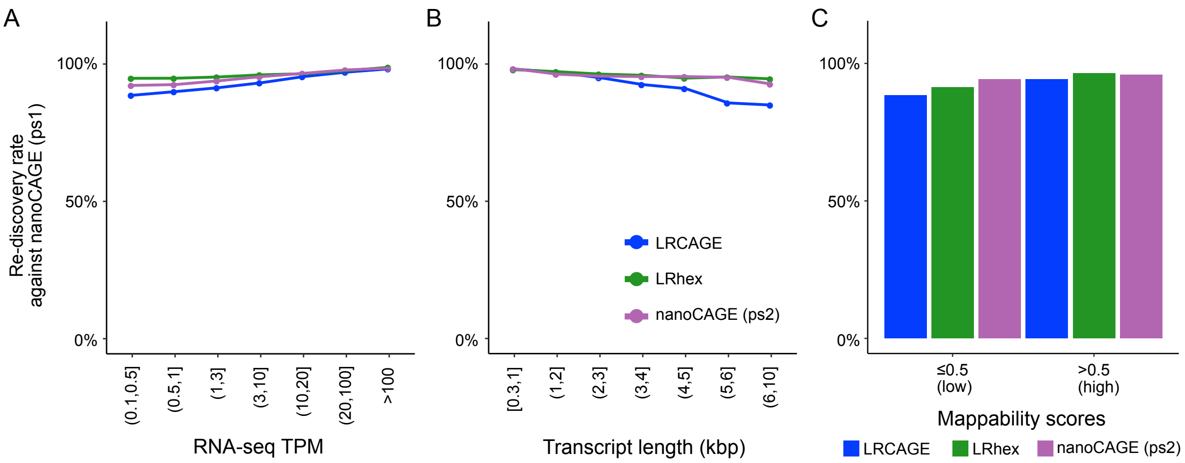

Supplemental Figure S4. Re-discovery rate against active GTSSs detected by nanoCAGE peaks as a function of expression levels, transcript length, and mappability scores. (A) expression levels. (B) transcript length. (C) mappability scores.

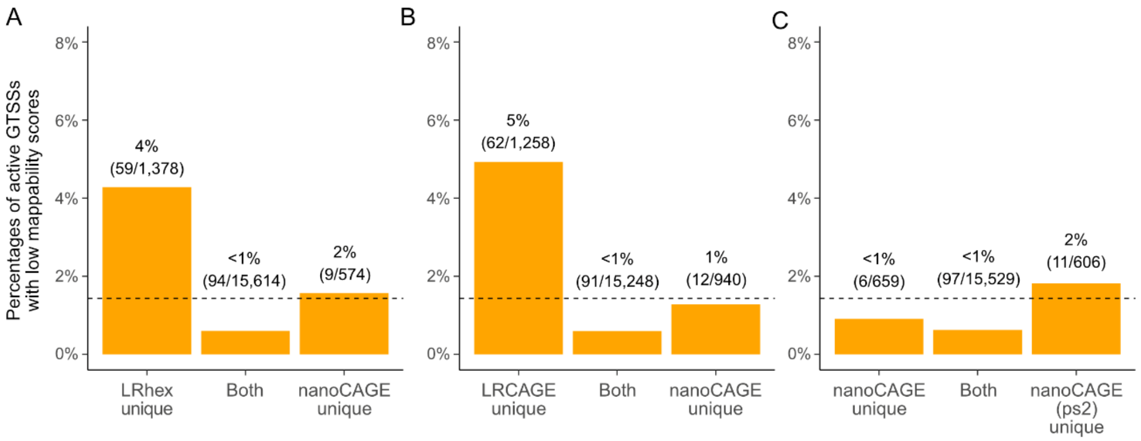

Supplemental Figure S5. Pairwise comparison of active GTSSs detected by LRhex, LRCAGE, and nanoCAGE peaks annotated with GTSSs with low mappability scores. (A) Percentages of active GTSSs with low mappability scores annotated with LRhex and nanoCAGE peak overlaps. (B) Percentages of active GTSSs with low mappability scores annotated with LRCAGE and nanoCAGE peak overlaps. (C) Percentages of active GTSSs with low mappability scores annotated with nanoCAGE pseudoreplicates (ps1 and ps2) peak overlaps. (A-C) A dashed line indicates percentages of active GTSSs with low mappability scores (1.4%).

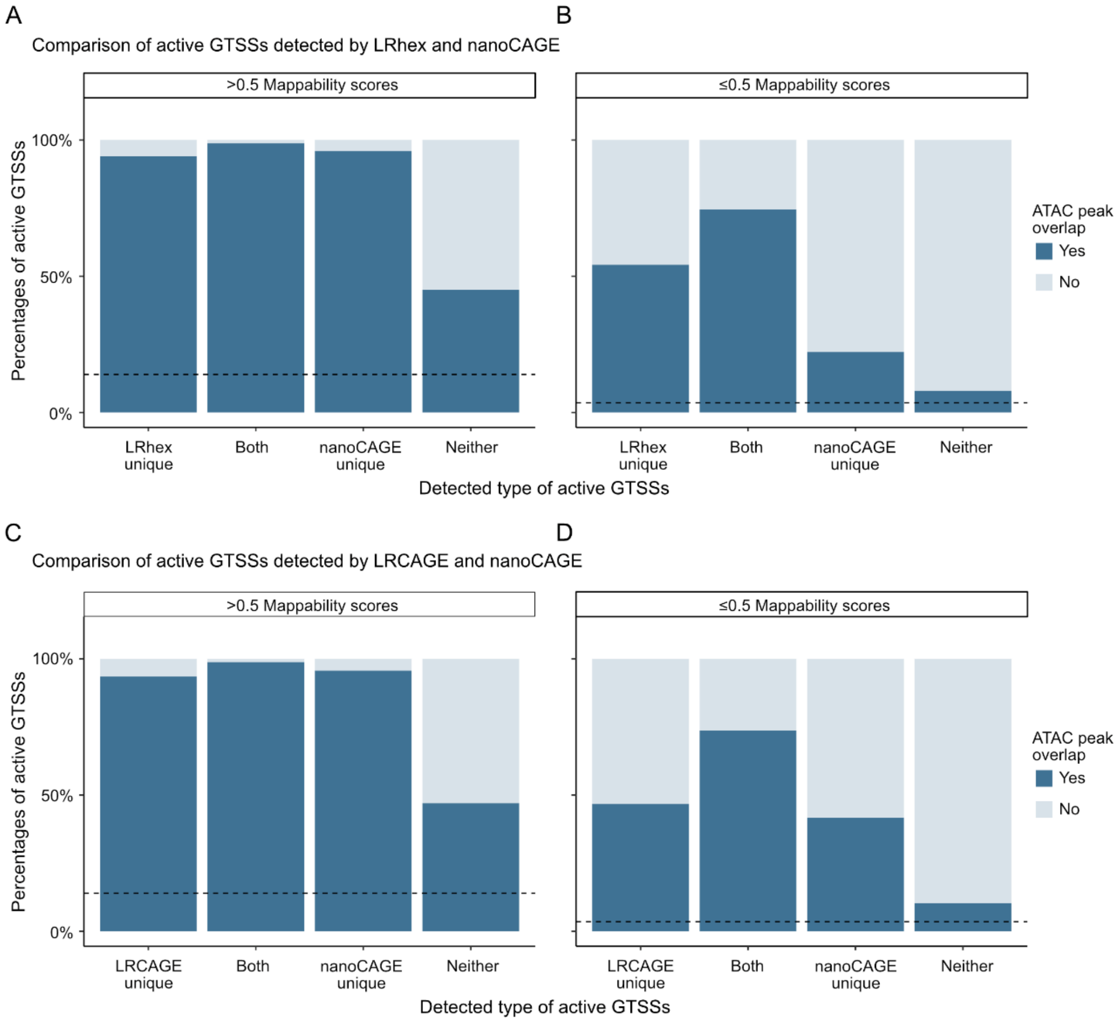

Supplemental Figure S6. Percentages of active GTSSs detected by LRhex, LRCAGE, and nanoCAGE peaks supported by ATAC peaks. (A) Proportions of active GTSSs with high mappability scores detected by LRhex and nanoCAGE peaks annotated with ATAC peak overlaps. (B) Proportions of active GTSSs with low mappability scores detected by LRhex and nanoCAGE peaks annotated with ATAC peak overlaps. (C) Proportions of active GTSSs with high mappability scores detected by LRCAGE and nanoCAGE peaks annotated with ATAC peak overlaps. (D) Proportions of active GTSSs with low mappability scores detected by LRCAGE and nanoCAGE peaks annotated with ATAC peak overlaps. (A-D) A dashed line indicates percentages of GTSSs with 0 TPM overlapped by ATAC peaks. The dashed line is at 14% (A, C) and at 4% (B, D).

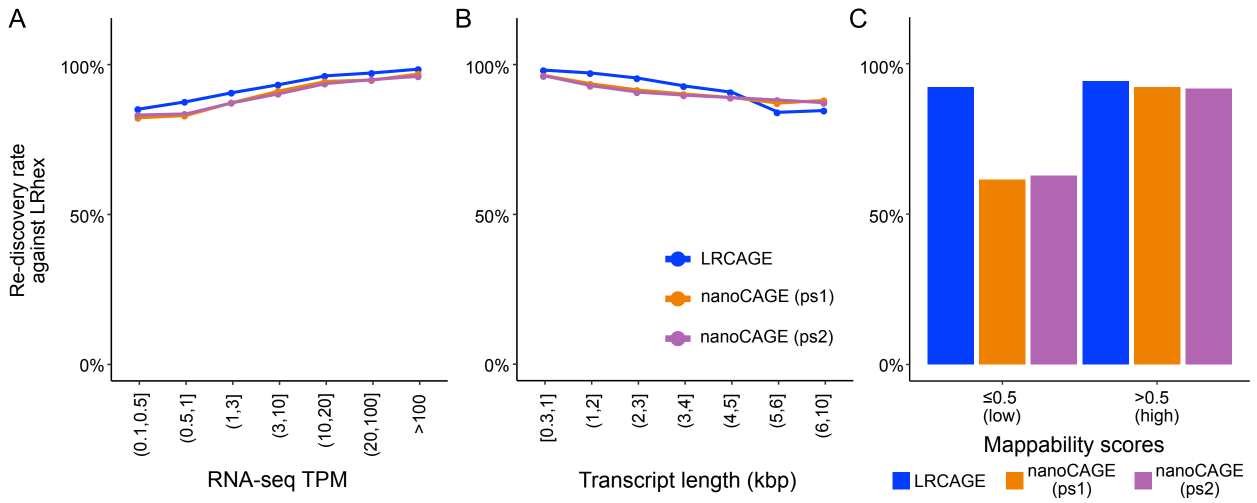

Supplemental Figure S7. Re-discovery rate against active GTSSs detected by LRhex peaks as a function of expression levels, transcript length, and mappability scores. (A) expression levels. (B) transcript length. (C) mappability scores. (A-C) nanoCAGE (ps1): nanoCAGE pseudo-replicate 1. nanoCAGE (ps2): nanoCAGE pseudo-replicate 2.

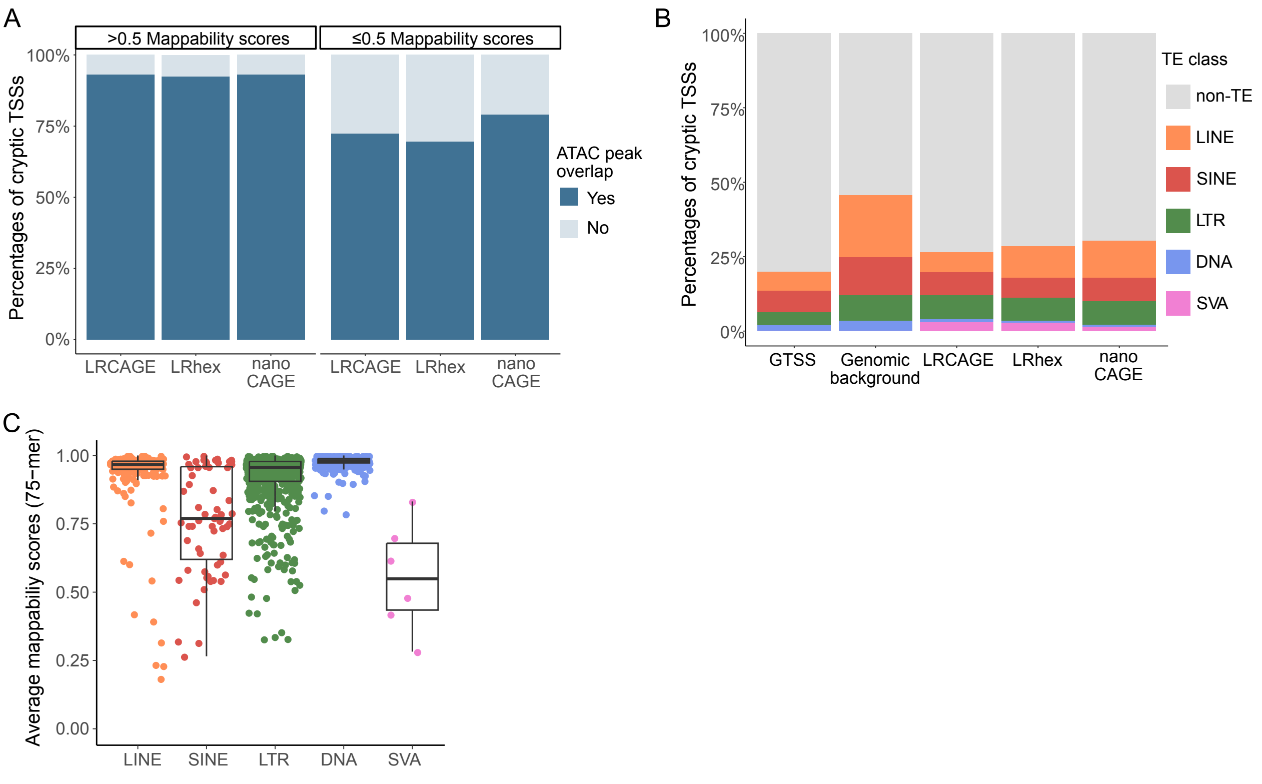

Supplemental Figure S8. Cryptic TSSs by their overlap with ATAC peaks and TEs. (A) Proportions of cryptic TSSs overlapping ATAC peaks annotated with mappability scores of cryptic TSSs. (B) Proportions of cryptic TSSs overlapping TEs. (C) Average mappability scores of TE subfamilies.

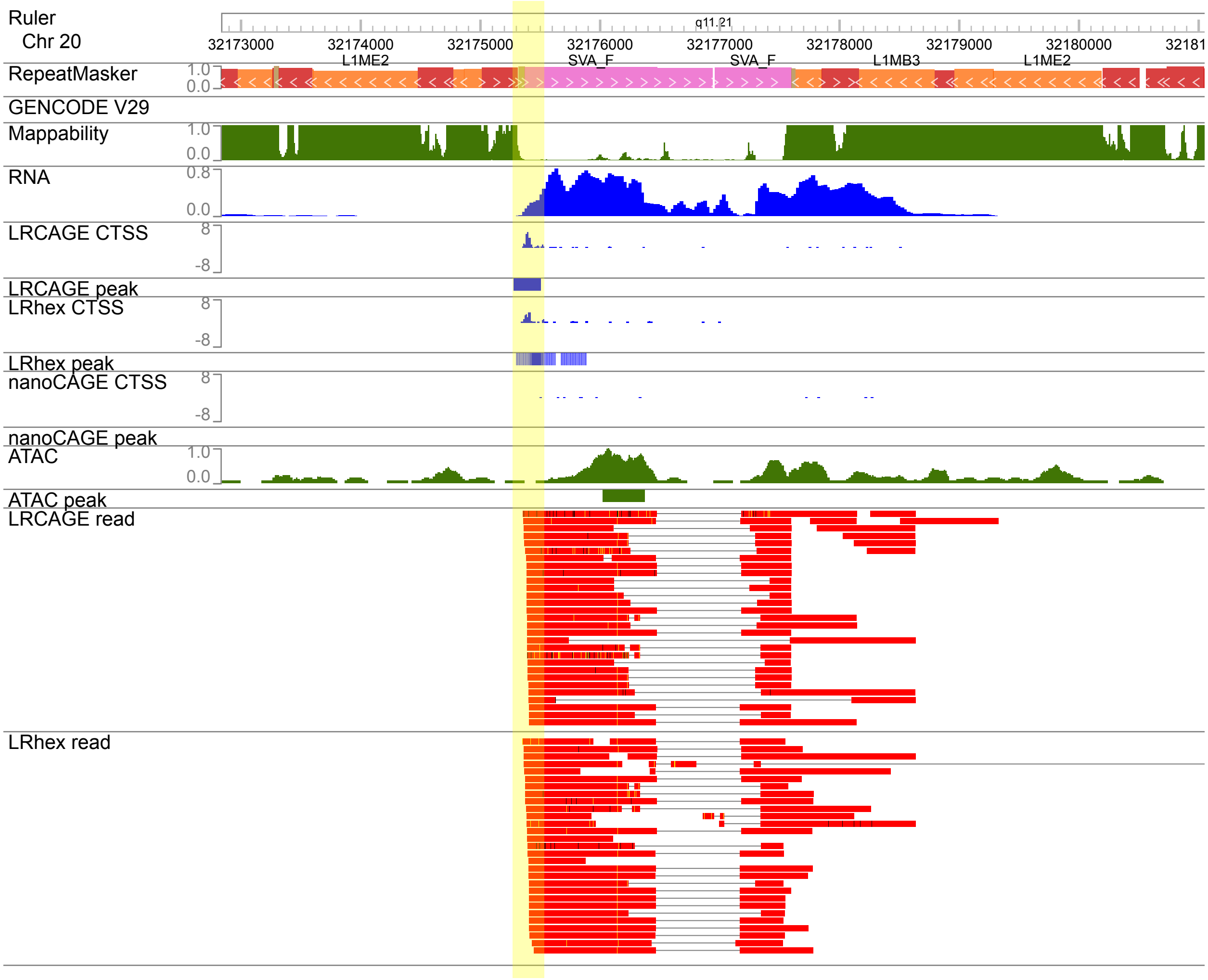

Supplemental Figure S9. Browser view of a SVA_F cryptic promoter (Chr 20:32,175,371-32,176,478) with LRCAGE and LRhex reads.

A

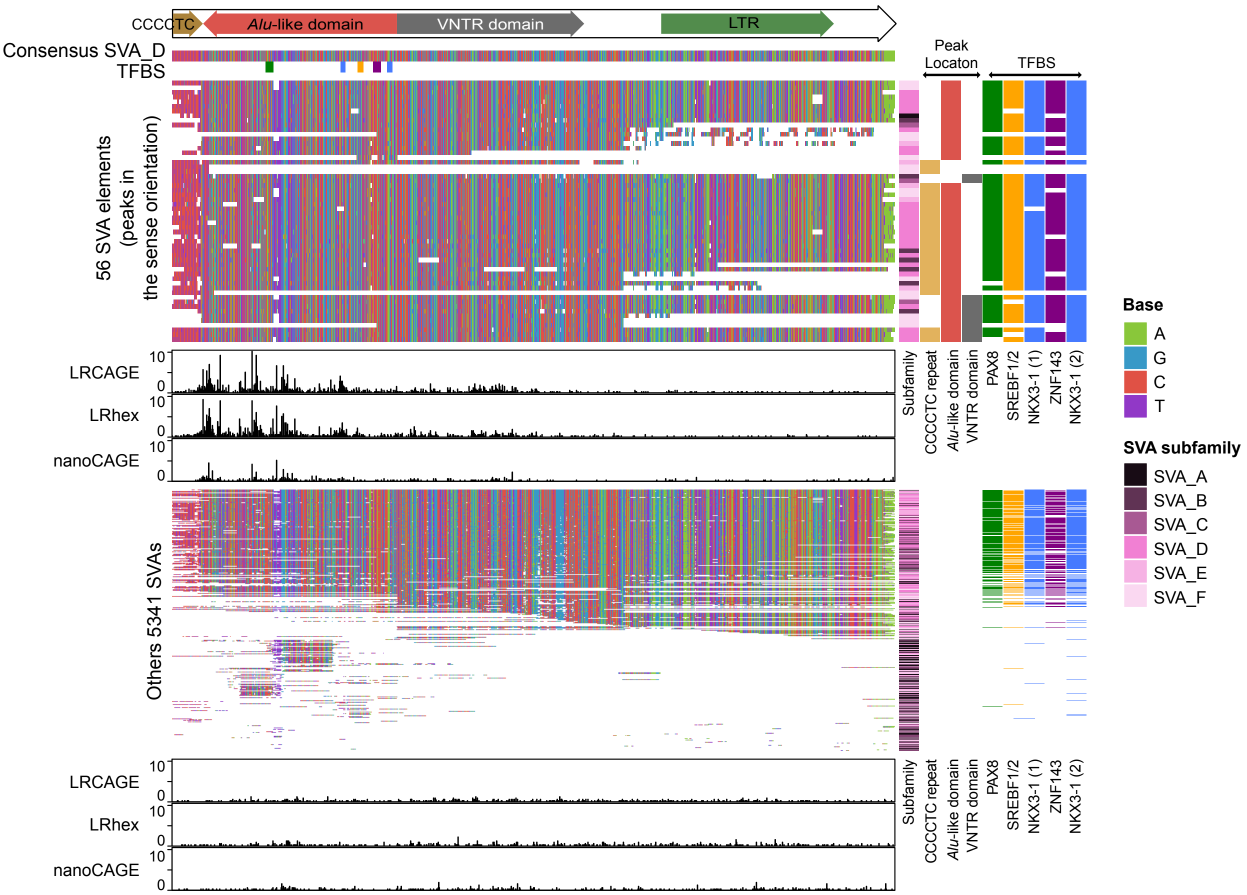

B

Motif: PAX8	SVA	With PAX8	W/O PAX8
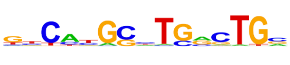	With peaks	48	8
	W/O peaks	1504	3404

Tetrachoric correlation: 0.54; Chi-square test p-value: <0.01

Motif: ZNF143	SVA	With ZNF143	W/O ZNF143
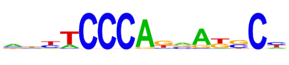	With peaks	44	12
	W/O peaks	1283	3625

Tetrachoric correlation: 0.50; Chi-square test p-value: <0.01

Motif: SREBF1, SREBF2	SVA	With SREBF1/2	W/O SREBF1/2
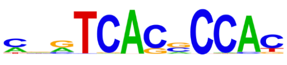	With peaks	47	9
	W/O peaks	1372	3536

Tetrachoric correlation: 0.54; Chi-square test p-value: <0.01

Motif: NKX3-1 (2)	SVA	With NKX3-1 (2)	W/O NKX3-1 (2)
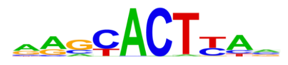	With peaks	52	4
	W/O peaks	1691	3217

Tetrachoric correlation: 0.60; Chi-square test p-value: <0.01

Motif: NKX3-1 (1)	SVA	With NKX3-1 (1)	W/O NKX3-1 (1)
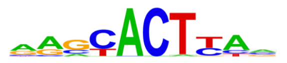	With peaks	49	7
	W/O peaks	1568	3340

Tetrachoric correlation: 0.55; Chi-square test p-value: <0.01

Supplemental Figure S10. Sequence context of promoter activities in SVA elements. (A) Heatmap of SVA elements aligned to consensus sequence of SVA_D subfamily. Presence of transcription factor binding sites (TFBSs) in SVA elements are annotated. Bar plot displays the total CTSS

signals across all SVA elements (only CTSS signals on the sense orientation of SVA elements are shown). (Top) 56 SVA elements having peaks in the sense orientation. SVA elements are sorted by length and peak location. (Bottom) 5,341 SVA elements without peaks in the sense orientation. SVA elements are sorted by length. (B) Contingency tables of SVA elements by TFBS and peak overlap. 4,908 SVA elements overlapping neither peaks nor CTSS signals were used as background. SREBF1 and SREBF2 motifs shared the binding site. NKX3-1 had two binding sites ~80bp apart.

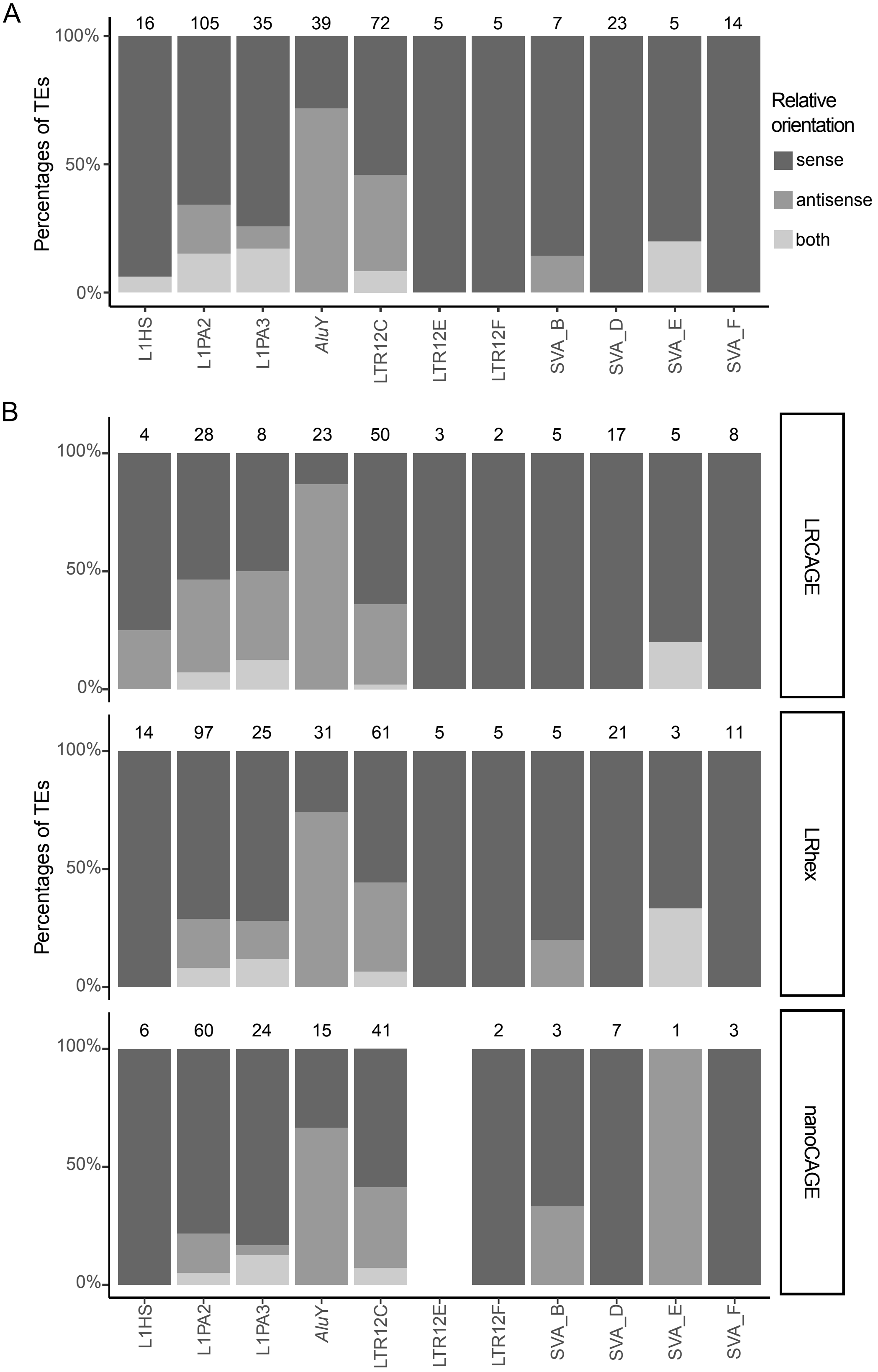

A
Percentages of TEs
100%
50%
0%
16 105 35 39 72 5 5 7 23 5 14
L1HS
L1PA2
L1PA3
AluY
LTR12C
LTR12E
LTR12F
SVA_B
SVA_D
SVA_E
SVA_F
Relative orientation
sense
antisense
both
B
LRCAGE
4 28 8 23 50 3 2 5 17 5 8
LRhex
14 97 25 31 61 5 5 5 21 3 11
nanoCAGE
6 60 24 15 41 2 3 7 1 3

Supplemental Figure S11. Relative orientation of TEs for overlapping cryptic TSSs. (A) Libraries combined. (B) By library type.

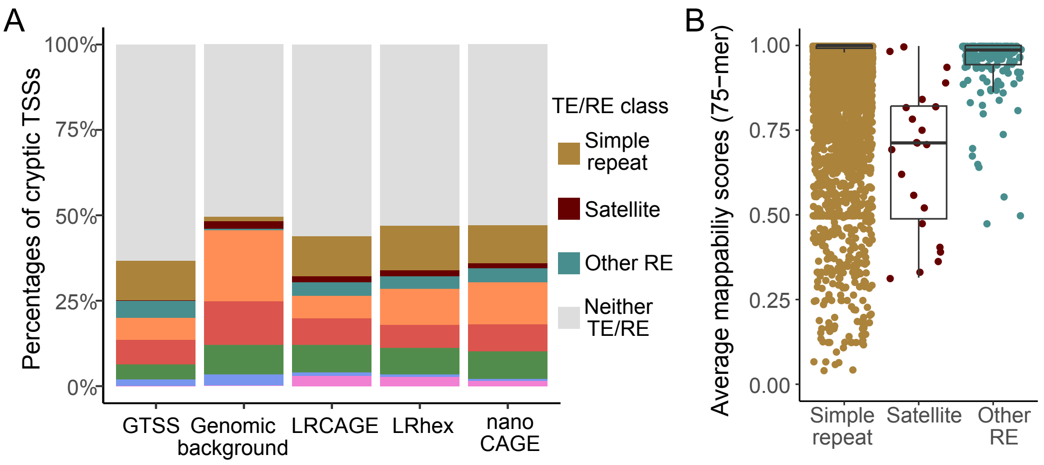

Supplemental Figure S12. Cryptic TSSs by their overlap with REs. (A) Proportions of cryptic TSSs overlapping TEs and REs. (B) Average mappability scores of RE subfamilies.

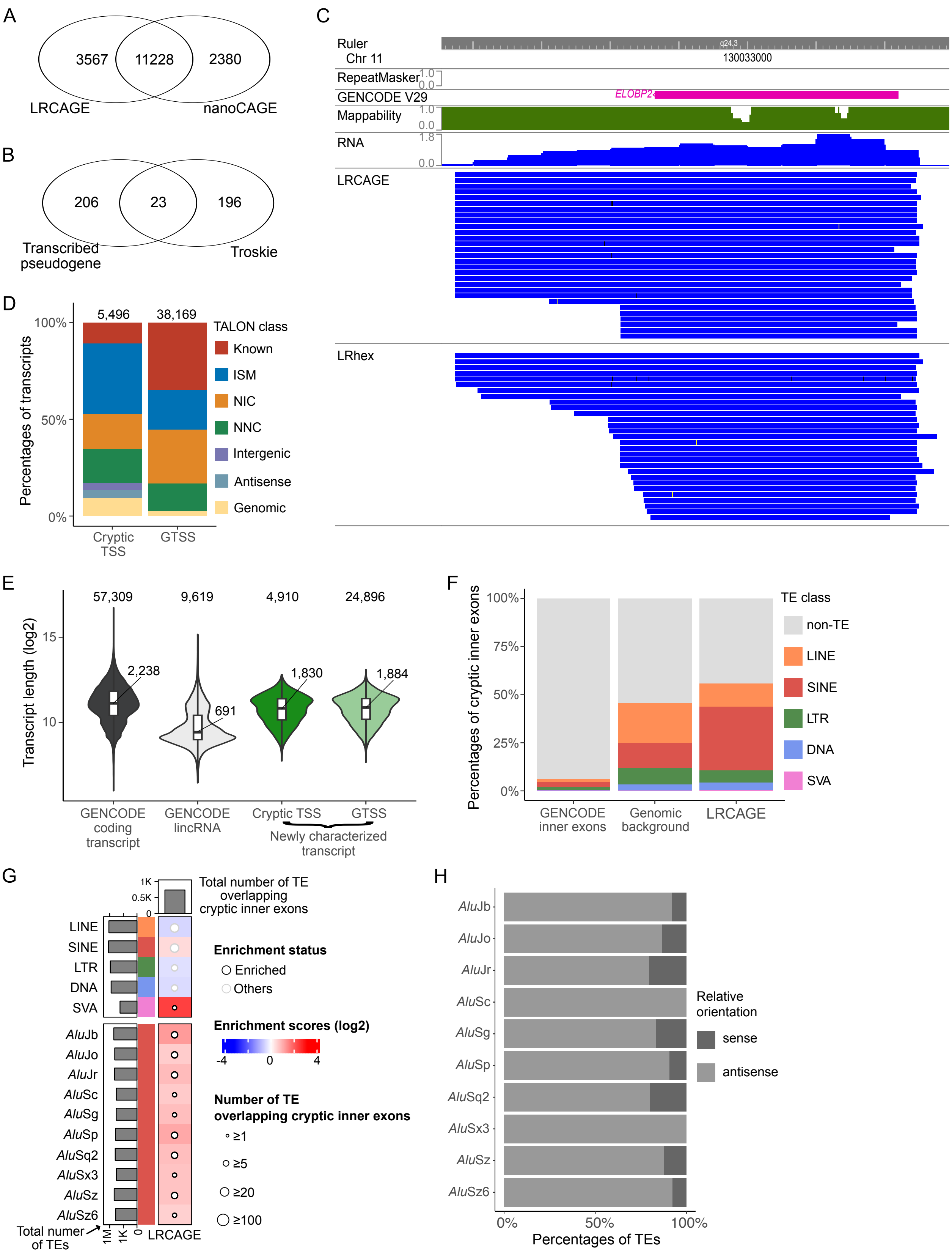
A
3567
11228
2380
LRCAGE
nanoCAGE
B
206
23
196
Transcribed pseudogene
Troskie
C
Ruler
Chr 11
130033000
RepeatMasker
GENCODE V29
ELOBP2
Mappability
RNA
LRCAGE
LRhex
D
Percentages of transcripts
100%
50%
0%
5,496
38,169
Cryptic TSS
GTSS
TALON class
Known
ISM
NIC
NNC
Intergenic
Antisense
Genomic
E
Transcript length (log2)
15
10
57,309
9,619
4,910
24,896
2,238
691
1,830
1,884
GENCODE coding transcript
GENCODE lincRNA
Cryptic TSS
GTSS
Newly characterized transcript
F
Percentages of cryptic inner exons
100%
75%
50%
25%
0%
GENCODE inner exons
Genomic background
LRCAGE
TE class
non-TE
LINE
SINE
LTR
DNA
SVA
G
1K
0.5K
0
Total number of TE overlapping cryptic inner exons
LINE
SINE
LTR
DNA
SVA
AluJb
AluJo
AluJr
AluSc
AluSg
AluSp
AluSq2
AluSx3
AluSz
AluSz6
Total numer of TEs
1M
1K
0
LRCAGE
Enrichment status
Enriched
Others
Enrichment scores (log2)
-4
0
4
Number of TE overlapping cryptic inner exons
≥1
≥5
≥20
≥100
H
AluJb
AluJo
AluJr
AluSc
AluSg
AluSp
AluSq2
AluSx3
AluSz
AluSz6
0%
50%
100%
Percentages of TEs
Relative orientation
sense
antisense

Supplemental Figure S13. Characteristics of transcripts profiled by LRCAGE data. (A) Venn diagram of LRCAGE peaks and nanoCAGE peaks. Peaks located within 200bp tolerance window in strand-sensitive manner are counted in the intersection area. (B) Venn diagram of transcriptionally-active pseudogenes in our data and Troskie's data. (C) Browser view at *ELOBP2* pseudogene. For visualization 30 reads from *ELOBP2* GTSS were randomly selected. (D) Proportions of LRCAGE transcripts by 5' end type annotated with TALON class. (E) Transcript length by GENCODE transcripts and newly characterized transcripts; Newly characterized transcripts are classified by their 5' end types: cryptic TSS-derived transcripts, GTSS-derived transcripts. (F) Proportions of cryptic inner exons overlapping TEs. (G) Cryptic inner exon enrichment heatmap by TE class and TE subfamily. Enriched TE subfamilies were defined as having ≥1.5 enrichment scores, ≥100 total TE elements, ≥10 TE elements overlapping cryptic inner exons. (H) Relative orientation of TEs for overlapping cryptic inner exons.

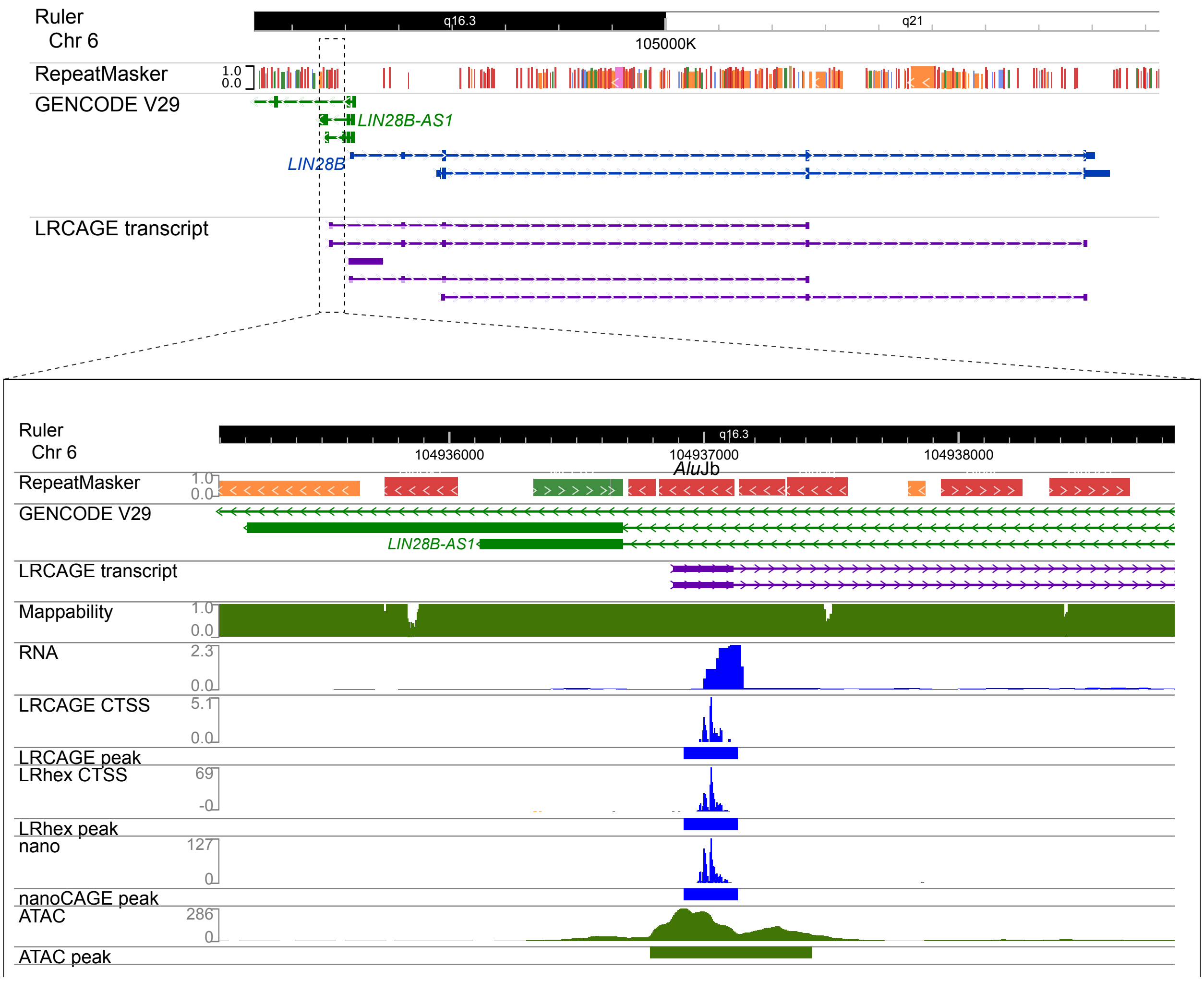

Supplemental Figure S14. *Alu*Jb-*LIN28B* transcripts detected by long-read CAGE data.

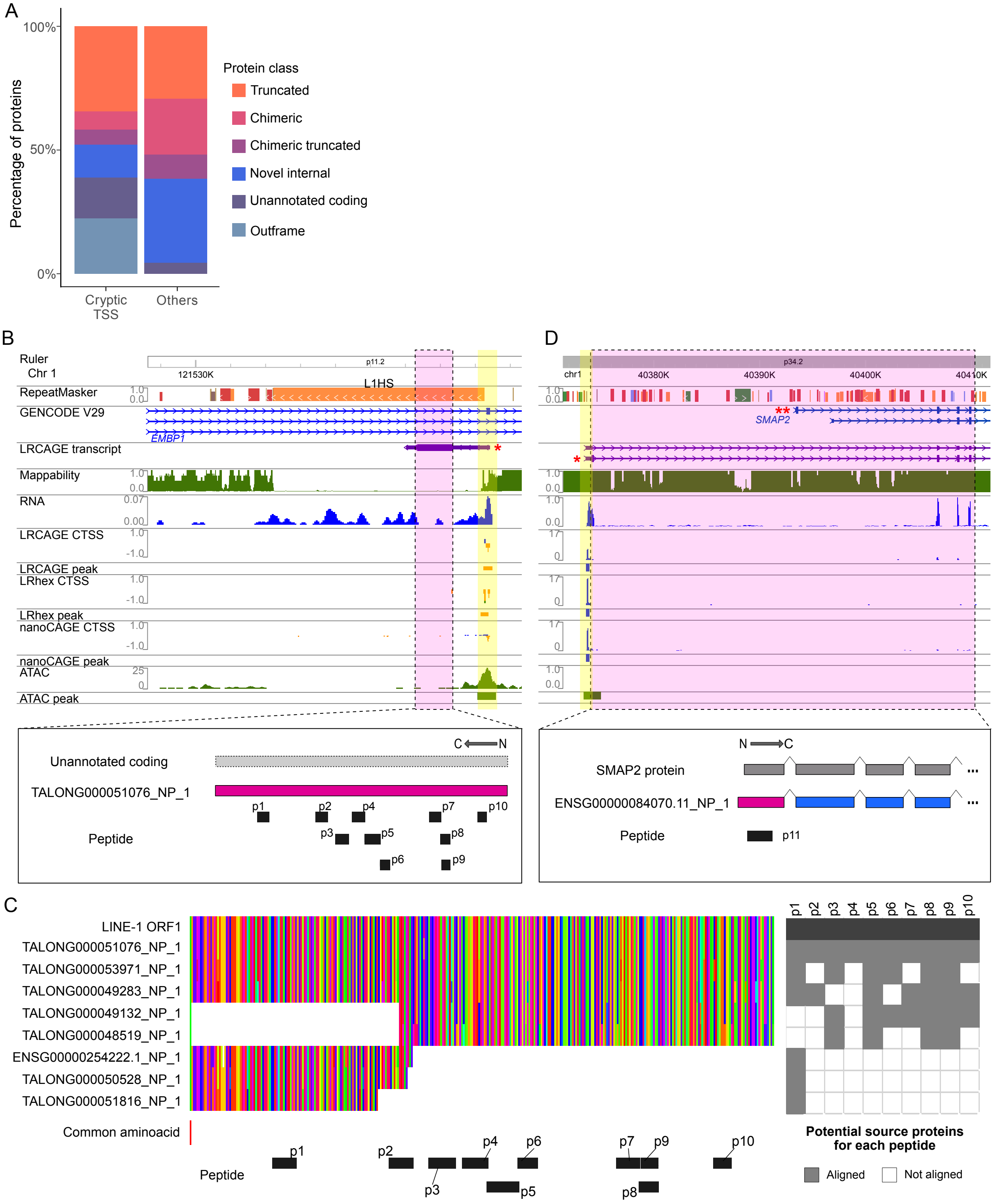

Supplemental Figure S15. Peptides from unannotated proteins in the LRCAGE proteome in H1299 whole cell lysate LC-MS/MS data. (A) Proportions of peptides from H1299 whole cell lysate MS data annotated with protein class by 5' end types of encoding transcripts. (B) Browser view

of TALONG000051076_NP_1 unannotated protein supported by 10 peptides from H1299 whole cell lysate MS data. (C) Multiple sequence alignment of TALONG000051076_NP_1, seven unannotated proteins containing any of 10 peptides aligned to TALONG000051076_NP_1, and LINE-1 ORF1. (D) Browser view of ENSG00000084070.11_NP_1 unannotated protein validated by one peptide from H1299 MS data. (B, D) Newly characterized transcripts encoding antigens are marked with a red asterisk (*). GENCODE transcripts encoding known proteins are marked with a double red asterisk (**). TSSs are marked with yellow bars. Protein coding regions are marked with pink bars. Peptide sequences are shown in Supplemental Table 4.

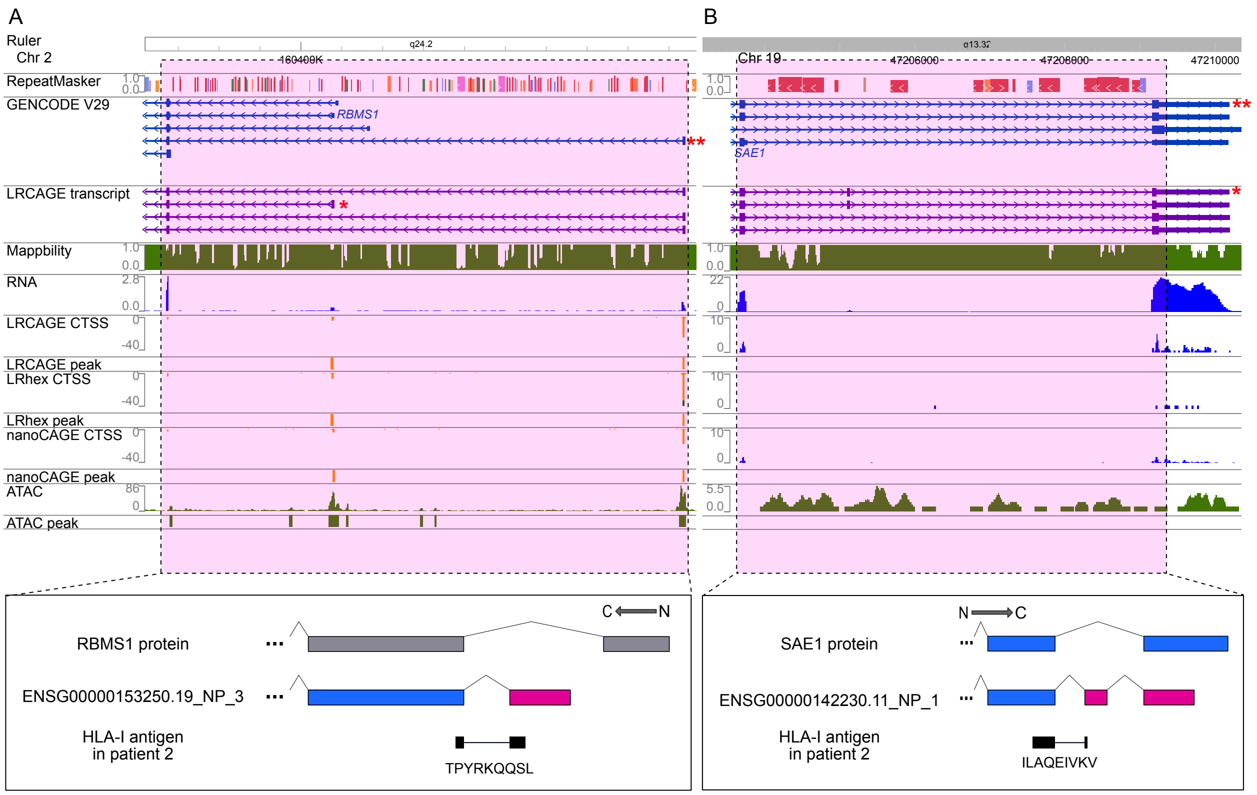

Supplemental Figure S16. Browser views of noncanonical antigens of two lung cancer patients. (A) Browser view of genomic locus producing an antigen, TPYRKQQSL. (B) Browser view of genomic locus producing an antigen, ILAQEIVKV. (A, B) Newly characterized transcripts encoding antigens are marked with a red asterisk (*). GENCODE transcripts encoding known proteins are marked with a double red asterisk (**). Protein coding regions are marked with pink bars.

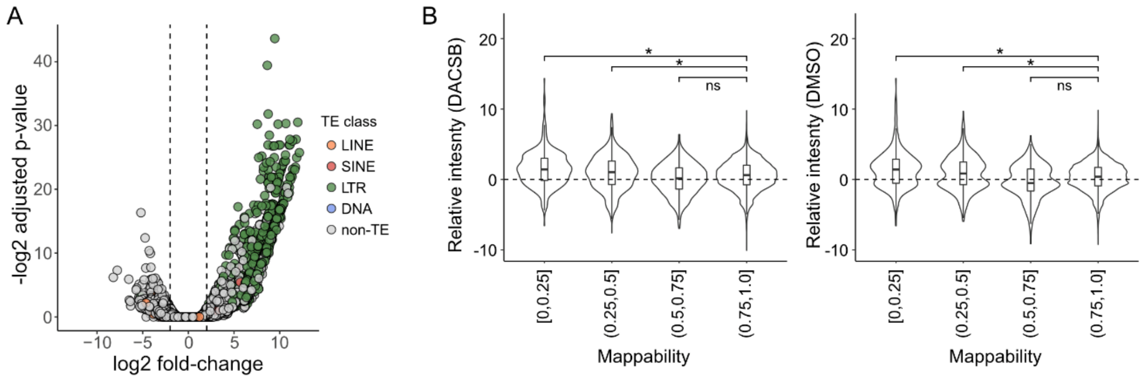

Supplemental Figure S17. Characteristics of consensus peaks. (A) Volcano plot of consensus peaks upon epigenetic treatment. Each dot represented a consensus peak and is annotated with TE class. (B) Relative peak intensity at consensus peak by LRCAGE data over nanoCAGE data as a function of mappability scores.

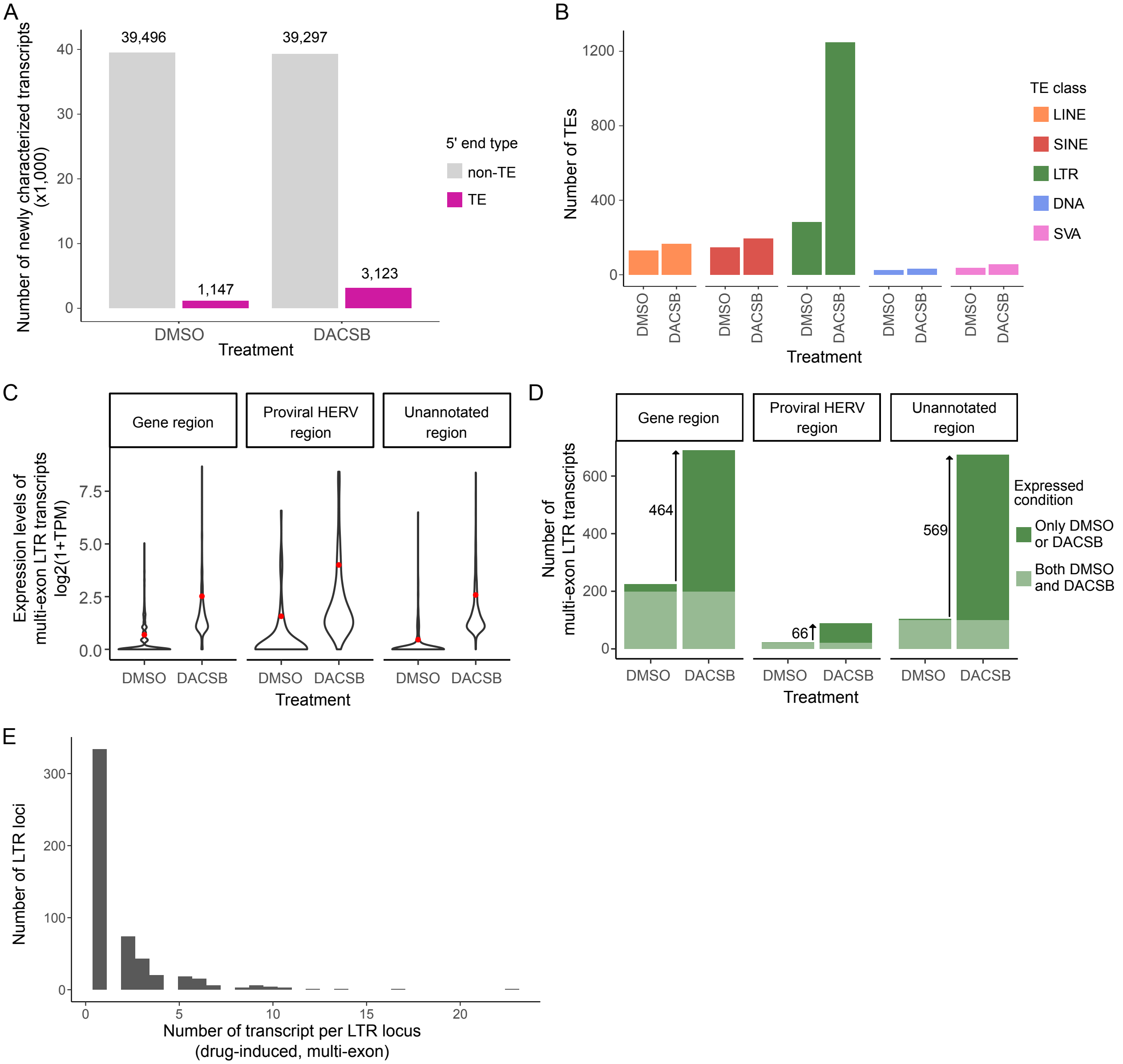

Supplemental Figure S18. Characteristics of newly characterized TE transcripts. (A) Number of newly characterized transcripts by 5' end types as a function of epigenetic treatment. (B) Number of TE elements producing newly characterized transcripts upon epigenetic treatment. (C) Expression levels of multi-exon LTR transcripts as a function of treatment condition using LRCAGE data. Number of transcripts (mean TPM in DMSO vs. DACSB conditions) - Gene region: 715 (0.6 vs. 4.9 TPM); Proviral HERV region: 89 (2.0 vs. 15.4 TPM); Unannotated region: 677 (0.4 vs. 5.1 TPM). Red point: mean value. (D) Number of newly characterized multi-exon LTR transcripts annotated with overlaps with GENCODE gene and proviral HERV annotations.

Transcripts expressed at ≥1 TPM are counted. Gene region: 225 (DMSO), 689 (DACSB); Proviral HERV region: 22 (DMSO), 88 (DACSB); Unannotated region: 104 (DMSO), 673 (DACSB). (E) Number of drug-induced multi-exon LTR transcript per LTR loci.

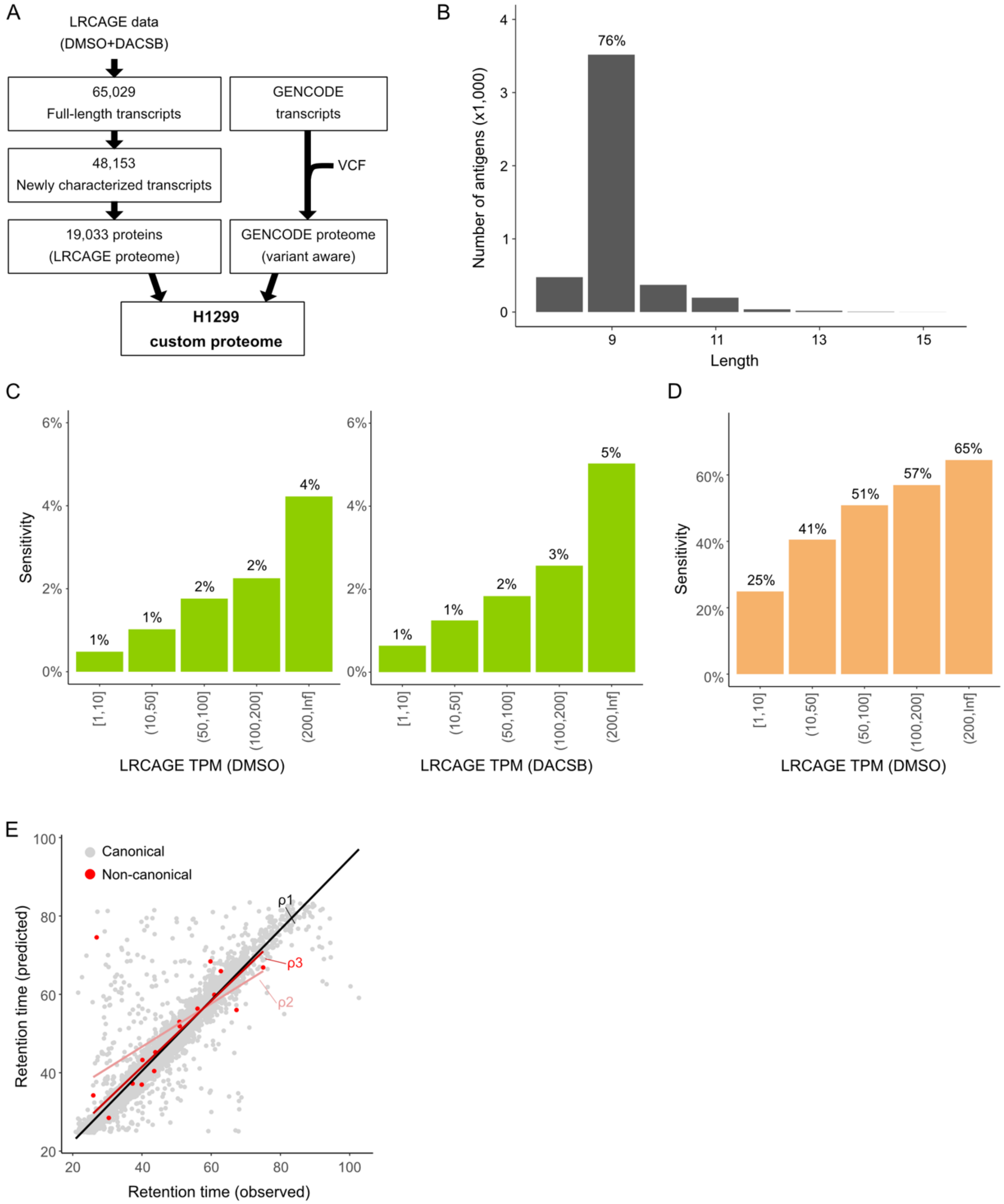

Supplemental Figure S19. Quality assessment of antigens from HLA-pulldown LC-MS/MS data. (A) Flowchart of preparing H1299 custom proteome using DMSO-treated and DACSB-treated H1299 LRCAGE data. (B) Size distribution of antigens. (C) Sensitivity of antigen predicted by NetMHC as a function of expression levels based on LRCAGE data. (D) Sensitivity of digested

peptide predicted by RPG as a function of expression levels based on LRCAGE data. (E) Correlations of observed predicted retention times and predicted retention times by DeepLC for antigens. ρ_1: correlation for canonical antigens (ρ_1=0.93, n: 4,593). ρ_2: correlation for noncanonical antigens (ρ_2=0.59, n: 16). ρ_3: correlation for noncanonical antigens after excluding one outlier (ρ_3=0.93, n: 15).

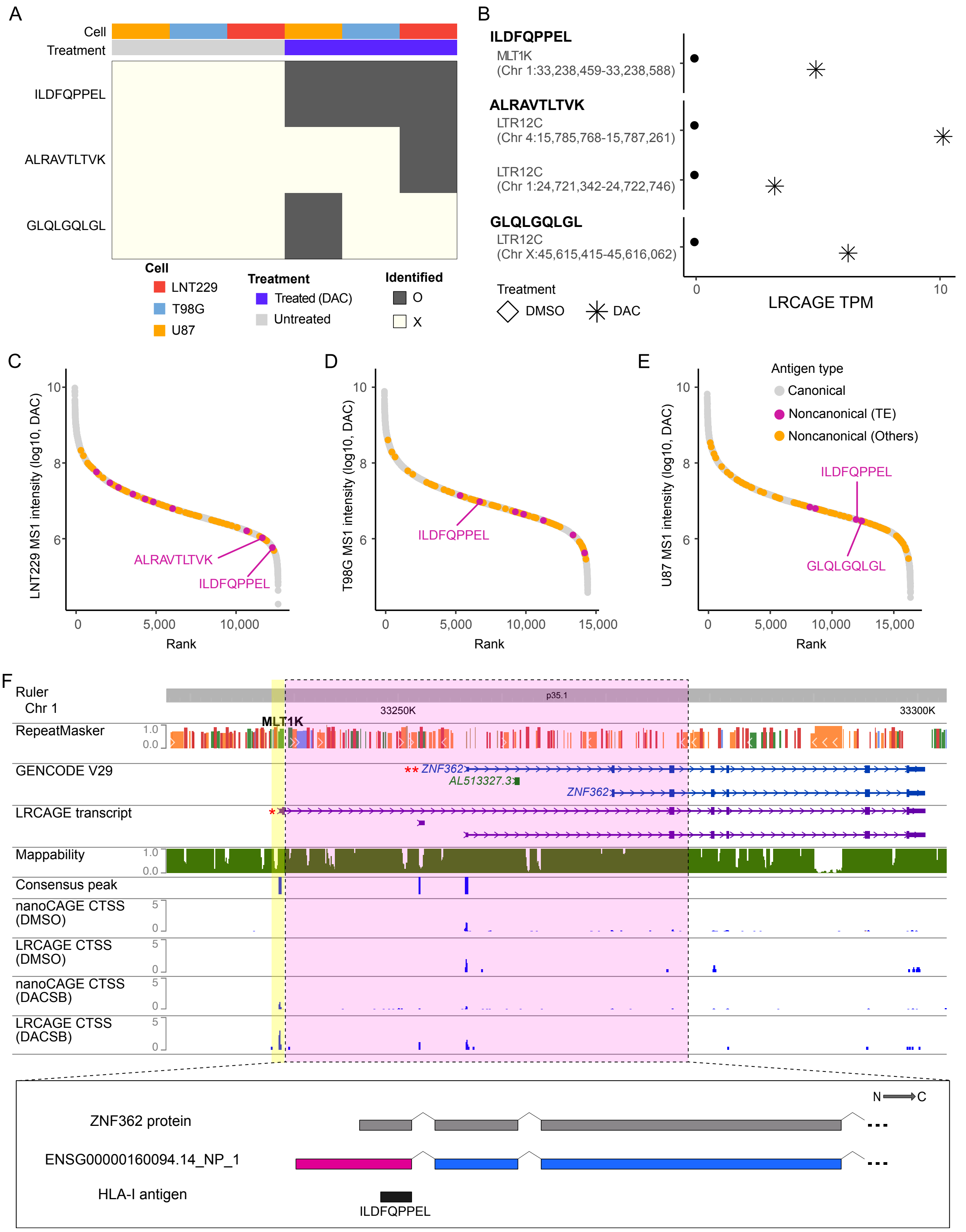

Supplemental Figure S20. Drug-induced noncanonical TE antigens in three glioblastoma cell lines. (A) Heatmap of noncanonical TE antigens unique to DAC-treated glioblastoma cell lines.

(B) Expression level of TE loci encoding drug-induced TE antigens. Expression levels are based on H1299 LRCAGE data. (C) MS1 intensity of antigens in LNT229 cells annotated with antigen types. (D) MS1 intensity of antigens in T98G cells annotated with antigen types. (E) MS1 intensity of antigens in U87 cells annotated with antigen types. (F) Browser view of a DAC-induced MLT1K transcript encoding ENSG00000160094.14_NP_1, producing ILDFQPPEL. Transcriptomics data is from H1299 cells. Newly characterized transcripts encoding antigens are marked with a red asterisk (*). GENCODE transcripts encoding known protein are marked with a double red asterisk (**). TSSs are marked with yellow bars. Protein coding regions are marked with pink bars.

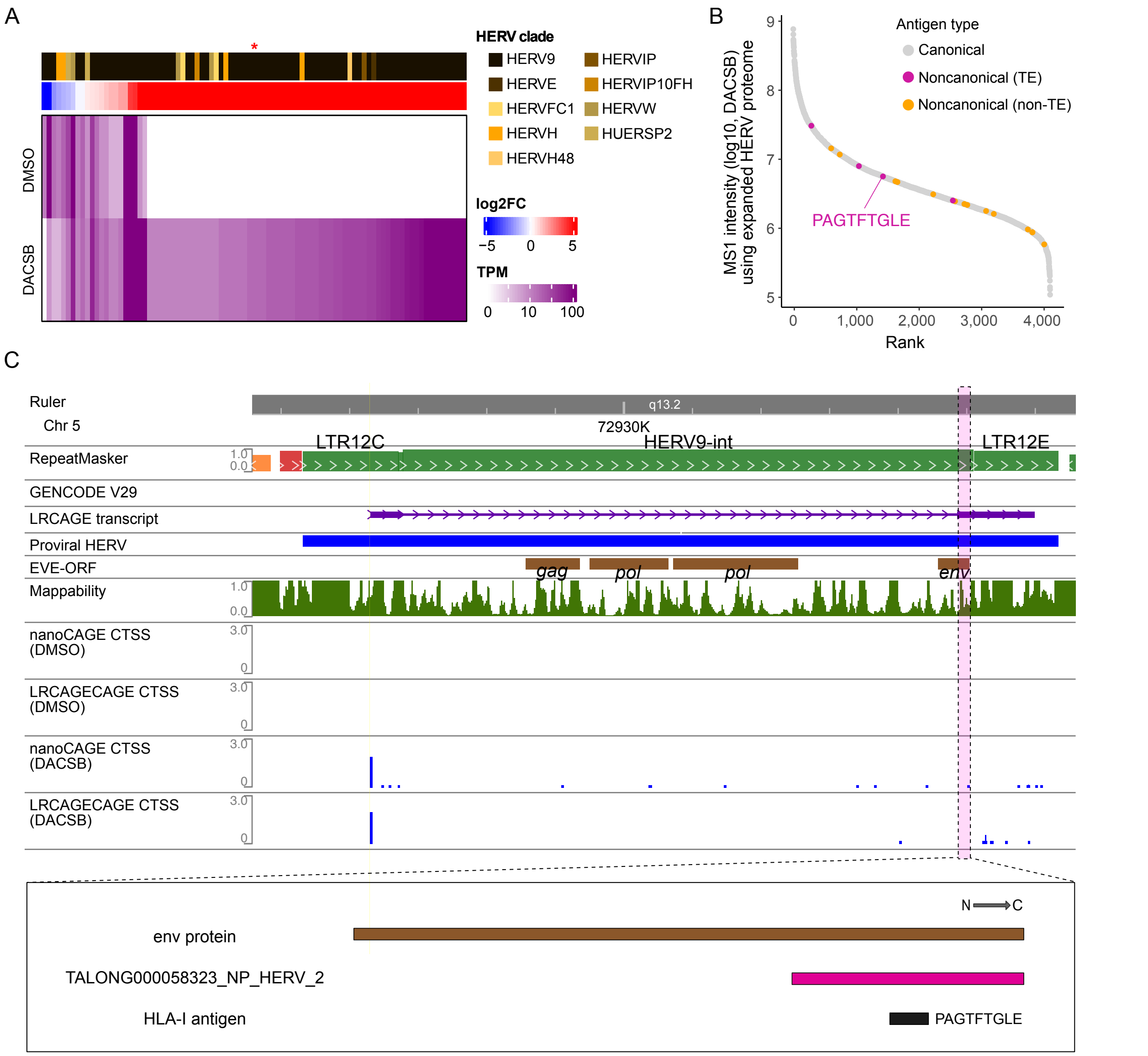

Supplemental Figure S21. A proviral HERV9 locus encoding *env*-derived antigen upon epigenetic treatment. (A) Expression level heatmap of HERV transcripts upon epigenetic treatment. An *env*-derived antigen coding transcript is marked with a red asterisk (*). (B) Browser view of a HERV9 transcript encoding an *env*-derived antigen, PAGTFTGLE. (C) MS1 intensity of an *env*-derived antigen, PAGTFTGLE.
