# Supplementary material for: Using long-read CAGE sequencing to profile cryptic-promoter-derived transcripts and their contribution to the immunopeptidome
Source: Genome Res. 2023 Dec;33(12):2143–55. doi: 10.1101/gr.277061.122 (PMC10760525; doi:10.1101/gr.277061.122)
Supplement: Supplement 3 [file Supplemental_Methods.pdf]

Supplemental Methods

Data processing for nanoCAGE and RNA-seq

For nanoCAGE data, UMIs and adaptor sequences were trimmed using tagdust (Lassmann 2015) and we filtered out unpaired read pair and read pair where either read length was shorter than 20bp using cutadapt (Martin 2011). Using the nanoCAGE library of 34 million reads as an input, we down-sampled into two smaller libraries of 4.1 million reads using 'seqtk sample' (https://github.com/lh3/seqtk) with different random number generator seeds, and we call the two smaller libraries "pseudo-replicates". For nanoCAGE and RNA-seq data, reads were aligned by STAR (v.2.5.4b) (Dobin et al. 2013) to the reference genome-guided by GENCODE annotation with the argument "--scoreDelOpen -1 --scoreDelBase -1 --scoreInsOpen -1 --scoreInsBase -1 --seedSearchStartLmax 15". Primary, non-supplementary, properly paired, and uniquely mapped reads were obtained. For nanoCAGE, we discarded reads with >3 soft-clip at 5' end of either read of pair. RNA-seq data of H1299 cells (Ghandi et al. 2019; Barretina et al. 2012, SRP186687) and 422 GTEx lung samples (Aguet et al. 2017, phs000424.v9.p2) were processed using the same pipeline.

SVA element analysis

Using needle (Rice et al. 2000; Needleman and Wunsch 1970), SVA element sequences were aligned to the SVA_D subfamily consensus sequence. The consensus sequence of SVA_D was chosen out of six SVA subfamilies because it had the most SVA elements with overlapping peaks. HOMER known motif analysis was used with the p-value cutoff of 1e-4 (Heinz et al. 2010). 56 SVA elements having peaks in the sense orientation were used as target. 4,908 SVA elements that did not overlap peaks or CTSS signals were used as background. Enriched TFBS motifs in *Alu*-like domain with corresponding transcription factors expressed at ≥1 TPM were used for

downstream analysis. Tetrachoric correlations and the Chi-square test were used, respectively, to determine the association between the presence of TFBS motifs and SVA promoters.

Enrichment analysis

Enrichment score (ES) of TE subfamily with cryptic TSSs was defined as follow.

$$ES = \frac{(\#\ of\ TEs\ per\ subfamily\ with\ cryptic\ TSSs)/(\#\ of\ TEs\ per\ subfamily)}{(\#\ of\ TEs\ with\ cryptic\ TSSs)/(\#\ of\ TEs)}$$

TE subfamilies were chosen for visualization based on three criteria: 1) at least 100 TEs, 2) ≥1.5 ES in at least one sample, and 3) at least 5 TEs having cryptic TSSs in at least one sample. We repeated the same calculation for TE class but included all TE class for visualization.

Similarly, ES of proviral HERV clade with up-regulated transcripts was defined as below.

$$ES = \frac{(\#\ of\ loci\ per\ clade\ with\ up-regulated\ transcripts)/(\#\ of\ loci\ per\ clade)}{(\#\ of\ loci\ with\ up-regulated\ transcripts)/(\#\ of\ loci)}$$

Mappability score calculation

We used GEM (Derrien et al. 2012) to calculate mappability scores by 75-mers using the reference genome as input.

Cancer-specific missense SNVs calling

Using GATK best practice for variant calling of SNV and INDEL, we identified SNVs using RNA-seq data of H1299 from CCLE project (Ghandi et al. 2019; Barretina et al. 2012, SRP186687) and normal lung samples from GTEx project (Aguet et al. 2017, phs000424.v9.p2). Cancer-specific missense SNVs were defined as missense SNVs in H1299 but not in >1% of normal lung samples using bcftools (v.1.10.2, Danecek et al. 2021).

Peptide analysis using LC-MS/MS and LC-MS3 data

H1299 whole lysate LC-MS/MS data (Choi et al. 2020, PXD016207) and whole lysate LC-MS3 data of LUAD cohorts (Gillette et al. 2020, PDC000153) were downloaded. HLA-pulldown LC-MS/MS data of 2 lung cancer patients (Chong et al. 2020, PXD013649) and glioblastoma cells (Shraibman et al. 2016, PXD003790) are downloaded. Different settings were used for MaxQuant as follows. H1299 whole lysate LC-MS/MS data: {type: "Standard", digestion mode: "Specific", enzyme: "Trypsin/P", peptide FDR 1%, protein FDR 1%}; CPTAC LUAD whole lysate LC-MS3 data: {type: "Reporter ion MS2" with the correction factor, digestion mode: "Specific", enzyme: "Trypsin/P", "LysC/P", peptide FDR 1%, protein FDR 1%}; HLA-pulldown LC-MS/MS data of 2 lung cancer patients, glioblastoma cells, and H1299 cells: Peptides that were potential contaminant or from reverse sequences were removed.

HLA-pulldown LC-MS/MS data generation

We followed the published HLA-I pulldown protocol (Bassani-Sternberg 2018; Marino et al. 2019) using 1 billion H1299 cells as input. We prepared two replicates for H1299 cells treated with DMSO and DACSB for each. LC-MS/MS analysis was carried out on an Orbitrap Fusion Lumos (Thermo Fisher Scientific, San Jose, CA) mass spectrometer coupled with a Dionex Ultimate 3000 RSLCnano HPLC (Thermo Fisher Scientific, San Jose, CA). The peptide separation was carried out on a Waters CSH C18 column (75 µm x 25 cm, 1.7 µm, Waters) at a flow rate of 0.3 µl/min and the following gradient: Time = 0–4 min, 2% B isocratic; 4–8 min, 2–10% B; 8–83 min, 10–25% B; 83–97 min, 25–50% B; 97–105 min, 50–98%. Mobile phase consisted of A, 0.1% formic acid; mobile phase B, 0.1% formic acid in acetonitrile. The instrument was operated in the data-dependent acquisition mode in which each MS1 scan was followed by Higher-energy collisional dissociation (HCD) MS/MS scan of as many precursor ions in 2 second cycle (Top Speed method). The mass range for MS1 was set to 300 to 1800 m/z with a resolution of 120,000 (200 m/z) and the automatic gain control (AGC) target set to 1,000,000 ions with a maximum fill time of 50 ms. For precursor selection, ions with charge state of 1 to 4 were selected. For MS/MS, the

selected precursors were fragmented in the Orbitrap using an isolation window of 1.6 m/z, a resolution of 30,000 (200 m/z), and a maximum fill time of 54 ms. Fragmentation energy in HCD MS/MS for charge state of 1 was set at higher level (32%) as opposed to 2 to 4 (27%) for more complete fragmentation. Dynamic exclusion was performed with a repeat count of 1, exclusion duration of 15 s, and a minimum MS ion count for triggering MS/MS set to 10000 counts.

HLA-I antigen and trypsin-digested peptide prediction

pVACtools (v2.0.2) (Hundal et al. 2020) was used ('pVACbind -e1 9') to predict antigens with NetMHC and NetMHCpan. We inferred HLA types of H1299 as HLA-A*32:01, HLA-A*24:02, HLA-B*40:02, HLA-C*02:02 with seq2HLA (v2.2, cutoff 0.1) and RNA-seq data (Boegel et al. 2012). For lung cancer patients, 14 HLA alleles that were prevalent in >5 % of Polish population and were available for NetMHC were used. For trypsin-digested peptide prediction, Rapid Peptides Generator was used (Maillet 2020).

Proviral HERV analysis

To prepare proviral HERV annotation, we used a custom script. Briefly, we combined annotation from Telescope (Bendall et al. 2019) with prototype class and from ERVmap (Tokuyama et al. 2018) with two flanking LTRs and one internal LTR element. For overlapping HERV loci, we chose HERV loci from Telescope. To detect EVE-ORF-derived antigens, we aligned antigens using to peptide sequences of EVE-ORFs from gEVE (Nakagawa and Takahashi 2016).

References

Aguet F, Brown AA, Castel SE, Davis JR, He Y, Jo B, Mohammadi P, Park YS, Parsana P, Segrè A V., et al. 2017. Genetic effects on gene expression across human tissues. *Nature* **550**: 204–213. doi:10.1038/nature24277.

Barretina J, Caponigro G, Stransky N, Venkatesan K, Margolin AA, Kim S, Wilson CJ, Lehár J, Kryukov G V, Sonkin D, et al. 2012. The Cancer Cell Line Encyclopedia enables predictive modelling of anticancer drug sensitivity. *Nature* **483**: 603–607. doi:10.1038/nature11003.

Bassani-Sternberg M. 2018. Mass spectrometry based immunopeptidomics for the discovery of cancer neoantigens. *Methods Mol Biol* **1719**: 209–221. doi:10.1007/978-1-4939-7537-2_14.

Bendall ML, De Mulder M, Iñiguez LP, Lecanda-Sánchez A, Pérez-Losada M, Ostrowski MA, Jones RB, Mulder LCF, Reyes-Terán G, Crandall KA, et al. 2019. Telescope: Characterization of the retrotranscriptome by accurate estimation of transposable element expression. *PLoS Comput Biol* **15**: 1–25. doi:10.1371/journal.pcbi.1006453.

Boegel S, Löwer M, Schäfer M, Bukur T, de Graaf J, Boisguérin V, Türeci Ö, Diken M, Castle JC, Sahin U. 2012. HLA typing from RNA-Seq sequence reads. *Genome Med* **4**. doi:10.1186/gm403.

Choi S, Ju S, Lee J, Na S, Lee C, Paek E. 2020. Proteogenomic Approach to UTR Peptide Identification. *J Proteome Res* **19**: 212–220. doi:10.1021/acs.jproteome.9b00498.

Chong C, Müller M, Pak HS, Harnett D, Huber F, Grun D, Leleu M, Auger A, Arnaud M, Stevenson BJ, et al. 2020. Integrated proteogenomic deep sequencing and analytics accurately identify non-canonical peptides in tumor immunopeptidomes. *Nat Commun* **11**. doi:10.1038/s41467-020-14968-9.

Danecek P, Bonfield JK, Liddle J, Marshall J, Ohan V, Pollard MO, Whitwham A, Keane T, McCarthy SA, Davies RM, et al. 2021. Twelve years of SAMtools and BCFtools. *Gigascience* **10**: 1–4. doi:10.1093/gigascience/giab008.

Derrien T, Estellé J, Sola SM, Knowles DG, Raineri E, Guigó R, Ribeca P. 2012. Fast computation and applications of genome mappability. *PLoS One* **7**. doi:10.1371/journal.pone.0030377.

Dobin A, Davis CA, Schlesinger F, Drenkow J, Zaleski C, Jha S, Batut P, Chaisson M, Gingeras TR. 2013. STAR: ultrafast universal RNA-seq aligner Alexander. *Bioinformatics* **29**: 15–21. doi:10.1093/bioinformatics/bts635.

Ghandi M, Huang FW, Jané-Valbuena J, Kryukov G V., Lo CC, McDonald ER, Barretina J, Gelfand ET, Bielski CM, Li H, et al. 2019. Next-generation characterization of the Cancer Cell Line Encyclopedia. *Nature* **569**: 503–508. doi:10.1038/s41586-019-1186-3.

Gillette MA, Satpathy S, Cao S, Dhanasekaran SM, Vasaikar S V., Krug K, Petralia F, Li Y, Liang WW, Reva B, et al. 2020. Proteogenomic Characterization Reveals Therapeutic Vulnerabilities in Lung Adenocarcinoma. *Cell* **182**: 200-225.e35. doi:10.1016/j.cell.2020.06.013.

Heinz S, Benner C, Spann N, Bertolino E, Lin YC, Laslo P, Cheng JX, Murre C, Singh H, Glass CK. 2010. Simple combinations of lineage-determining transcription factors prime cis-regulatory elements required for macrophage and B cell identities. *Mol Cell* **38**: 576–589. doi:10.1016/j.molcel.2010.05.004.

Hundal J, Kiwala S, McMichael J, Miller CA, Xia H, Wollam AT, Liu CJ, Zhao S, Feng YY, Graubert AP, et al. 2020. PVACtools: A computational toolkit to identify and visualize cancer neoantigens. *Cancer Immunol Res* **8**: 409–420. doi:10.1158/2326-6066.CIR-19-0401.

Lassmann T. 2015. TagDust2: A generic method to extract reads from sequencing data. *BMC Bioinformatics* **16**. doi:10.1186/s12859-015-0454-y.

Maillet N. 2020. Rapid Peptides Generator: fast and efficient in silico protein digestion. *NAR Genomics Bioinforma* **2**: 1–10. doi:10.1093/nargab/lqz004.

Marino F, Chong C, Michaux J, Bassani-Sternberg M. 2019. High-throughput, fast, and sensitive

immunopeptidomics sample processing for mass spectrometry. In *Methods in Molecular Biology*, Vol. 1913 of, pp. 67–79 doi:10.1007/978-1-4939-8979-9_5.

Martin M. 2011. Cutadapt removes adapter sequences from high-throughput sequencing reads. *EMBnet.journal*. doi:10.14806/ej.17.1.200.

Nakagawa S, Takahashi MU. 2016. gEVE: a genome-based endogenous viral element database provides comprehensive viral protein-coding sequences in mammalian genomes. *Database* **2016**: 1–8. doi:10.1093/database/baw087.

Needleman SB, Wunsch CD. 1970. A general method applicable to the search for similarities in the amino acid sequence of two proteins. *J Mol Biol* **48**: 443–453. doi:10.1016/0022-2836(70)90057-4.

Rice P, Longden L, Bleasby A. 2000. EMBOSS: The European Molecular Biology Open Software Suite. *Trends Genet* **16**: 276–277. doi:10.1016/S0168-9525(00)02024-2.

Shraibman B, Kadosh DM, Barnea E, Admon A. 2016. Human leukocyte antigen (HLA) peptides derived from tumor antigens induced by inhibition of DNA methylation for development of drug-facilitated immunotherapy. *Mol Cell Proteomics* **15**: 3058–3070. doi:10.1074/mcp.M116.060350.

Tokuyama M, Kong Y, Song E, Jayewickreme T, Kang I, Iwasaki A. 2018. ERVmap analysis reveals genome-wide transcription of human endogenous retroviruses. *Proc Natl Acad Sci U S A* **115**: 12565–12572. doi:10.1073/pnas.1814589115.
